# The mechanism of a formaldehyde-sensing transcriptional regulator

**DOI:** 10.1038/srep38879

**Published:** 2016-12-09

**Authors:** Katie J. Denby, Jeffrey Iwig, Claudine Bisson, Jodie Westwood, Matthew D. Rolfe, Svetlana E. Sedelnikova, Khadine Higgins, Michael J. Maroney, Patrick J. Baker, Peter T. Chivers, Jeffrey Green

**Affiliations:** 1 Department of Molecular Biology and Biotechnology, University of Sheffield, Sheffield, S10 2TN, UK; 2 Department of Biochemistry and Molecular Biophysics, Washington University School of Medicine in St. Louis, St. Louis, MO, 63110, USA; 3 Department of Chemistry, University of Massachusetts-Amherst, Amherst, MA 01003, USA; 4 Departments of Biosciences and Chemistry, Durham University, Durham, DH1 3LE, UK

## Abstract

Most organisms are exposed to the genotoxic chemical formaldehyde, either from endogenous or environmental sources. Therefore, biology has evolved systems to perceive and detoxify formaldehyde. The *frmRA(B)* operon that is present in many bacteria represents one such system. The FrmR protein is a transcriptional repressor that is specifically inactivated in the presence of formaldehyde, permitting expression of the formaldehyde detoxification machinery (FrmA and FrmB, when the latter is present). The X-ray structure of the formaldehyde-treated *Escherichia coli* FrmR (*Ec*FrmR) protein reveals the formation of methylene bridges that link adjacent Pro2 and Cys35 residues in the *Ec*FrmR tetramer. Methylene bridge formation has profound effects on the pattern of surface charge of *Ec*FrmR and combined with biochemical/biophysical data suggests a mechanistic model for formaldehyde-sensing and derepression of *frmRA(B)* expression in numerous bacterial species.

All organisms must adapt to withstand the detrimental effects of toxic chemicals. Some of these toxic compounds, such as reactive oxygen and nitrogen species are generated endogenously, as a result of metabolic processes; others are natural or synthetic products that are present in the environment. Many of these chemicals have broad reactivity, which presents biological systems with a challenge to specifically sense and then respond to their presence before the cell suffers irreversible damage. Formaldehyde is a toxic electrophilic chemical that is generated endogenously by many organisms^1^,^2^. For example, formaldehyde is generated: (i) as an intermediate in methylotrophic metabolism; (ii) in the degradation of glycine, either by the glycolytic byproduct methylgloxal or by Fenton chemistry; (iii) in the degradation of heme, during iron acquisition by some Gram-positive bacteria; (iv) by lipid peroxidation of sugars; (v) by the demethylation of histones; (vi) as a product of methylated-DNA repair by AlkB; and (vii) by the action of *N-*methyltryptophan oxidase (SolA)^2^,^3^,^4^. Consequently, biological systems are often exposed to both endogenous and exogenous sources of formaldehyde.

Formaldehyde mediates its toxic effects by chemically modifying vital cell components, including DNA and proteins, thereby leading to cellular dysfunction. Formaldehyde-mediated genotoxicity is caused by the formation DNA-DNA and DNA-protein cross-links, as well as covalent DNA monoadducts^1^,^2^,^5^,^6^,^7^. In addition, formaldehyde is able to covalently modify proteins, inhibiting their functions^7^,^8^. The life-threatening damage caused by the broad chemical reactivity of formaldehyde has driven the evolution of mechanisms to detoxify formaldehyde and counteract its detrimental effects^2^. In order to regulate expression of these detoxification systems, it is imperative to maintain specific response-regulators in the cell that can perceive the presence of formaldehyde and induce protective systems before significant damage to vital cell components occurs.

The starting point for the work reported here was the observation that formaldehyde is generated when the model bacterium *Escherichia coli* adapts to the presence of the alternative electron acceptor trimethylamine-*N*-oxide^9^. An inability to respond (by induction of the *frmRAB* operon) to this endogenous formaldehyde challenge resulted in growth inhibition, rather than growth promotion, when anaerobic *E. coli* cultures were provided with trimethylamine-*N*-oxide^9^. The *frmRAB* operon codes for: a regulator, FrmR (*Ec*FrmR); a formaldehyde dehydrogenase, FrmA; and an *S*-formylglutathione hydrolase, FrmB^10^. In *E. coli*, and many other organisms, formaldehyde in the cytosol reacts with the major reductant glutathione, yielding *S*-hydroxymethylglutathione, which is then oxidized by FrmA to *S*-formylglutathione^11^,^12^. In *E. coli S*-formylglutathione is hydrolyzed to formate and glutathione by the product of the final gene of the *frmRAB* operon, FrmB, or by a second, less-efficient hydrolase YeiG^11^; some bacteria, such as the closely related *Salmonella enterica* serovar Typhimurium, lack *frmB* but possess *yeiG*. Thus, the proteins coded by the *frmRAB* operon and *yeiG* permit bacteria to sense and detoxify formaldehyde.

The first gene of the *E. coli frmRAB* operon is the regulatory protein *Ec*FrmR. *Ec*FrmR is a member of the CsoR/RcnR family of transcriptional repressors^13^,^14^. Most of the characterized members of this family are metal-ion sensors; the properties of Cu(I) (CsoR, RicR), Ni(II) (InrS) and Ni(II)/Co(II) (RcnR, DmeR) sensors in diverse bacterial species have been reported, as well as those of the sulfite/sulfide sensor CstR^15^,^16^,^17^,^18^,^19^. Amino acid sequence alignments of the metal-sensing members of the family revealed characteristic amino acid signatures (known as the W-X-Y-Z fingerprint) that are associated with metal-binding (Fig. 1a). The availability of structure-function data now allows the W-X-Y-Z fingerprint to be considered spatially, centered about the absolutely conserved Cys residue (position X, Fig. 1a; residue 35 in RcnR and FrmR), rather than as a primary sequence motif^17^,^20^. In addition to the absolute conservation at position X, there is a highly conserved His residue at position Y (Fig. 1a). RcnR and InrS use all four positions to co-ordinate their cognate metal, and in addition RcnR uses the *N*-terminal residue, which is hereafter regarded as spatially part of position W, for metal-coordination^17^,^21^. CsoR binds Cu(I) via the X-Y-Z positions (Fig. 1a). Amino acid residue variation at different positions within the fingerprint is implicated in signal specificity by coupling metal-coordination preferences to the allosteric network connecting the metal- and DNA-binding residues of the proteins^21^. Whilst the Ni(II)/Co(II) and Cu(I)-responsive family members are relatively well-characterized, little is known about signal perception and DNA-binding mechanisms of the remaining proteins (CstR and FrmR), although the Cys residues at positions X and Z in *S. aureus* CstR, and Pro2 and Cys35 (positions W and X) of *Salmonella enterica* serovar Typhimurium FrmR (hereafter *Sty*FrmR) have been implicated in sulfide and formaldehyde sensing, respectively (Fig. 1a)^19^,^22^,^23^.

*Sty*FrmR retains 3 of the metal-binding ligands (His3, Cys35 and His60, with Glu instead of His64) that are present in the paralogous *Salmonella* Ni(II)-binding regulator RcnR (Fig. 1a)^22^. Recent *in vitro* studies showed that *Sty*FrmR binds Co(II), Cu(I) and Zn(II), but the binding affinities were weaker than those of the biological sensors of these metal ions in *Salmonella*, such that *Sty*FrmR was outcompeted by CueR, RcnR, ZntR and ZurR for their cognate metals^22^. Thus *in vivo, Sty*FrmR was able to act as a formaldehyde-responsive transcription regulator, but was unable to act as a metal ion sensor^22^. Further work showed that formaldehyde-sensing by *Sty*FrmR is specific, direct and requires two residues of the W-X-Y-Z fingerprint (the *N*-terminal Pro at position W and Cys35 at position X; Fig. 1)^23^. The crystal structure of a metal-sensing *Sty*FrmR variant (*Sty*FrmR(E64H)), created by site-directed mutagenesis, revealed that it consists of a homotetrameric disc with a surface-exposed positively-charged region that is predicted to participate in DNA-binding^23^. Hence, the fold of *Sty*FrmR resembles other members of the CsoR/RcnR family^24^,^25^.

Amino acid sequence alignment of the *E. coli* K-12 FrmR (*Ec*FrmR) and *Sty*FrmR proteins reveals strong conservation in the first 63 amino acids (67% identical, 89% similar) but weaker conservation thereafter (21% identical; 39% similar; Fig. 1b). There is limited conservation between the two proteins within the W-X-Y-Z fingerprint. Both have Cys and His at positions X and Y, as well as Pro at residue 2 (position W). However, *Ec*FrmR differs with Ser not His at residue 3 (a component of position W; note that the equivalent of His3 is involved in metal-binding in RcnR and InrS proteins) and Thr not Glu at position Z. Notably, *Ec*FrmR possesses an additional non-conserved Cys residue (Cys70) (Fig. 1b). The differences between *Sty*FrmR and *Ec*FrmR along with the biological imperative to mount an effective response to endogenous sources of formaldehyde, as evidenced by the observation that the *frmRAB* operon was essential for adaptation of *E. coli* to growth on trimethylamine-*N*-oxide, prompted an investigation of the *Ec*FrmR protein^9^. This is worthy of investigation because, although the activities of detoxifying enzymes, such as FrmA and FrmB, have been established, the mechanism(s) used by regulatory proteins to perceive and respond to formaldehyde are poorly understood. Hence, the aim of the work reported here was to provide new insight into the mechanism used by biological systems to sense the presence of the highly reactive and toxic chemical, formaldehyde. *In vivo* and *in vitro* data show that *Ec*FrmR senses formaldehyde directly, with no metal-dependence, via the formation of inter-subunit methylene bridges between adjacent Pro2 and Cys35 residues. This formaldehyde-mediated cross-linking remodels the surface of the tetrameric *Ec*FrmR disc resulting in de-repression of *frmRAB* expression by promoting disassociation of the *frmRAB* promoter (P_*frm*_)-*Ec*FrmR complex.

## Results and Discussion

### *Ec*FrmR is a formaldehyde sensor

Herring and Blattner showed that *Ec*FrmR is necessary for repression of the *frmRAB* promoter (P_*frm*_) in *E. coli*, and that the operon is induced in the presence of formaldehyde^10^. Similarly, *Sty*FrmR responds to formaldehyde, but not acetaldehyde or organic alcohols^23^. A P_*frm*_-*frmR-lacZ* reporter fusion containing ~500 bp upstream of the *frmA* open reading frame, so as to include *frmR*, was constructed to determine the range of molecules that de-repress *frmRAB* expression (Table S1). β-Galactosidase activity (proxy for *frmRAB* expression) was induced 28-fold in the presence of 700 μM formaldehyde (Fig. 2a). The role of *Ec*FrmR in this regulation was confirmed by mutation of codon six of *frmR* to a stop codon (P_*frm*_-*frmR*_stop_*-lacZ*), whereupon constitutive high expression was observed, consistent with *Ec*FrmR-mediated repression of *frmRAB* in the absence of formaldehyde (Fig. 2a). The signal specificity of *frmRAB* induction was assessed by culturing the reporter strain in the presence of different aldehydes (Fig. 2b). Many of these compounds have been found to induce intracellular damage, suggesting that they can cross the cell membrane, and hence they could be directly or indirectly perceived by cytosolic FrmR^26^,^27^,^28^. Formaldehyde induced the highest β-galactosidase activity, but acetaldehyde, methylglyoxal and glyoxal also induced expression, albeit to lesser extents (Fig. 2b). This contrasts with *Sty*FrmR which did not respond to acetaldehyde (in the same type of experiment), suggesting sequence differences between the two proteins around the sensory site could affect selectivity. Bulky aldehydes, such as furaldehyde and tribromoacetaldehyde, were unable enhance *lacZ* expression. Overall, these responses suggest that, although induction of *frmRAB* is not absolutely specific, formaldehyde is by far the most effective inducer, consistent with the specificity of the detoxification machinery (FrmA and FrmB/YeiG). However, it is possible that induction results from *Ec*FrmR responding to the formation of *S*-hydroxymethylglutathione in the cytoplasm rather than formaldehyde *per se*.

### *Ec*FrmR binds directly to the *frmRAB* promoter (P_*frm*_) to repress transcription

The effect of signal molecules on DNA-binding affinity and transcriptional regulation provides a sensitive measure of allosteric effectiveness. Bio-Layer Interferometry (BLItz) measurements were used to investigate interactions between the *Ec*FrmR protein and *frmRAB* promoter DNA (P_*frm*_). Under these conditions a K_d_ for *Ec*FrmR binding to immobilized P_*frm*_ DNA was ~220 nM, determined from an overall on-rate constant (*k*_f_) of ~13000 M^−1^ s^−1^ and an overall off-rate constant (*k*_r_) of ~0.003 s^−1^ at 20 °C (Fig. 3a; Table 1). This K_d_ value is similar to that reported for *Sty*FrmR (~100 nM)^22^, but should be considered as an upper limit because the potential effects of low level metal contamination and protein oxidation (see below) on DNA-binding are unknown. Nevertheless, this interaction was specific because the K_d_ for *Ec*FrmR binding at an unrelated promoter DNA fragment (*E. coli ydhY*) was only ~3600 nM (Table 1). Pre-treatment of *Ec*FrmR protein with excess formaldehyde for 3 min essentially abolished specific binding to P_*frm*_ (Fig. 3a). Exposure of the pre-formed P_*frm*_-*Ec*FrmR binary complex to increasing concentrations of formaldehyde resulted in disassociation of the complex (Fig. 3b). The P_*frm*_–*Ec*FrmR disassociation curves were fitted to a single exponential function. Disassociation of the P_*frm*_-*Ec*FrmR-complex exhibited a linear dependence on formaldehyde concentration, with a rate constant of ~4 M^−1^ s^−1^ at 20 °C (Fig. 3c; Table 1). Accordingly, *in vitro* transcription reactions showed that synthesis of the *frmRAB* transcript was inhibited in the presence of *Ec*FrmR and that this inhibition was relieved when *Ec*FrmR was treated with formaldehyde (Fig. 3d). *Ec*FrmR did not affect transcription from the *E. coli ndh* promoter, indicating that the effects of *Ec*FrmR and formaldehyde on *frmRAB* transcription were specific (Fig. 3d). Therefore, the *in vivo* and *in vitro* data showed that *Ec*FrmR is a repressor of *frmRAB* expression that responds directly to formaldehyde by disassociation of the P_*frm*_–*Ec*FrmR complex, allowing the expression of the formaldehyde detoxification system.

### Properties of isolated *Ec*FrmR

Liquid chromatography-mass spectrometry (LC-MS) showed that *Ec*FrmR lacked an *N*-terminal methionine (confirmed by *N*-terminal amino acid sequencing) and had the expected monomeric molecular mass of 10186.50 Da. *Ec*FrmR eluted from a calibrated size exclusion chromatography column at a volume indicative of a tetramer and this assignment was confirmed by analytical ultracentrifugation, which yielded a mass of 44.9 kDa (Fig. S1a). Thus, like other members of the CsoR/RcnR family, *Ec*FrmR is a homotetramer. The isolated protein was metal-free as judged by inductively coupled plasma mass spectrometry (ICP-MS) analysis and reaction with 5, 5′-dithiobis(2-nitrobenzoic acid) (DTNB) indicated the presence of 1.86 ± 0.21 reactive thiols per monomer. However, the number titratable thiol groups decreased to 1.02 ± 0.08 per monomer upon aerobic storage (>72 h), suggesting that protein can adopt an oxidized form with two disulfide bonds per tetramer during prolonged exposure to molecular oxygen.

**Figure 1:**
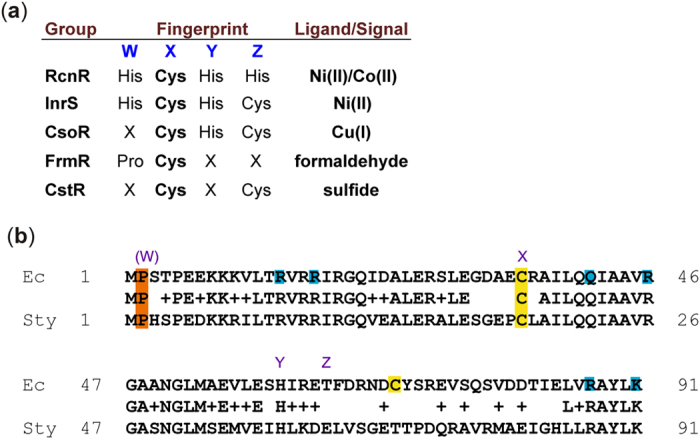
Amino acid fingerprints associated with signal perception by members of the CsoR/RcnR family and amino acid sequences of *Ec*FrmR and *Sty*FrmR. (**a**) Amino acid residues in the W-X-Y-Z fingerprint of CsoR/RcnR family proteins and the signals perceived by the indicated proteins. (**b**) Alignment of the *E. coli* (Ec) and *S. enterica* serovar Typhimurium (Sty) FrmR proteins. Identical (single letter code) and similar (+) residues, Pro2 (brown background), Cys35 and Cys70 (yellow background) are indicated. Residues of the W-X-Y-Z fingerprint (blue font) are indicated. Position W is shown to incorporate both Pro2 and Ser/His3 (as indicated by parentheses; see text for details). Residues on blue backgrounds have been implicated in DNA-binding in other CsoR/RcnR family members.

As *Sty*FrmR is able to bind Zn(II)^22^, the interaction of *Ec*FrmR with Zn(II) was also examined. Zn(II) addition resulted in changes in intrinsic fluorescence at 304 nm (Fig. S1b), with saturation at 4 Zn(II) atoms per *Ec*FrmR tetramer. In contrast, titrations with Mn(II) resulted in a linear non-specific binding response that did not saturate even at a [Mn(II)]:[*Ec*FrmR tetramer] ratio of 19. The titration with Zn(II) suggested a sub-micromolar binding affinity, so a competition assay with mag-fura2 (*K*_d_ = 61.9 nM) was used to measure *K*_Zn_ (Fig. S1c). The data were best fit to a model of two pairs of two independent sites (*K*_1_ = *K*_2_ and *K*_3_ = *K*_4_), with *K*_1_ = 3.1 ± 0.3 nM and *K*_3_ = 219 ± 19 nM. The value for *K*_1_ and *K*_2_ represents an upper limit as the theoretical curve determined for *K*_1_ and *K*_2_ = 0.31 nM (i.e. 10-fold tighter binding at the first two sites) is not well-distinguished from the experimental data (Fig. S1c). This model is consistent with different stepwise binding affinities reported for other family members^20^. The Zn(II) affinity of *Ec*FrmR was significantly weaker under non-reducing conditions (*K*_dapp_ = 146 ± 32 nM), suggesting that the thiolate of a Cys residue (most likely Cys35 at the X position of the W-X-Y-Z metal binding motif: Fig. 1) is important for Zn(II) binding. The affinity of *Ec*FrmR for Zn(II) is similar to that reported for *Sty*FrmR (0.17 ± nM), so Zn(II) could contribute to formaldehyde-sensing by both these proteins^22^. To gain structural insight into Zn(II) coordination by *Ec*FrmR, Zn-saturated *Ec*FrmR tetramers were analyzed by X-ray absorption spectroscopy (XAS). X-ray absorption near edge structure (XANES) data suggested a five-coordinate Zn-site (Fig. S1d). However, the best fit to the extended X-ray absorption fine structure (EXAFS) data was obtained with a tetrahedral model in which Zn(II) is coordinated by a His N atom at 2.00 Å (probably His60; see above), one thiolate ligand at 2.27 Å (probably Cys35; see above), an N/O ligand at 2.00 Å and an additional ligand from the solvent (Br^-^ or Cl^-^ from the buffer; Fig. S1d; Table S2). Thus, *Ec*FrmR likely binds Zn(II) via three amino acid side-chains leaving the fourth co-ordination position free, possibly to participate in binding formaldehyde, by analogy to substrate coordination in Zn-dependent alcohol dehydrogenases^29^.

The ability of FrmR proteins to bind Zn(II) raises the possibility that formaldehyde sensing and/or DNA-binding is enhanced by metal ions. Therefore, the effect of Zn(II) on the ability of *Ec*FrmR to bind to DNA (P_*frm*_) was assessed by BLItz (Table 1). Loading the *Ec*FrmR tetramer with 4 molar equivalents of Zn(II) increased the K_d_ for binding at P_*frm*_ ~2-fold as a result of a decrease in the rate constant for DNA-binding (Table 1). However, the disassociation rate constant of the pre-formed Zn(II)-loaded P_*frm*_–*Ec*FrmR complex in response to formaldehyde exposure was ~7-fold lower than that observed in the absence of Zn(II), suggesting that Zn(II) blocks amino acid residues required for formaldehyde sensing, as determined by the XAS experiments, and would be antagonistic to the deployment of the detoxification machinery. Therefore, it was concluded that formaldehyde-sensing and DNA-binding are not metal- (Zn(II)-) dependent and, based on the affinity of *Ec*FrmR (and *Sty*FrmR) for Zn(II), it is likely that Zn(II) could only inhibit FrmR activity *in vivo* under conditions when Zn(II) homeostasis is severely perturbed (Fig. S2; Table 1)^22^.

### Identification of *Ec*FrmR residues necessary for formaldehyde sensing

Site-directed mutagenesis of the amino acids of the W-X-Y-Z fingerprint has revealed the importance of these residues for the function of CsoR/RcnR family proteins (Fig. 1a). Recently, Pro2 (position W) and Cys35 (position X) of *Sty*FrmR have been shown to be required for formaldehyde-responsiveness *in vivo* and *in vitro*, whereas Glu64 (position Z) was not^23^. Therefore, to identify *Ec*FrmR residues necessary for formaldehyde sensing, site-directed mutation of the P_*frm*_-*frmR-lacZ* construct (see above) was undertaken. β-Galactosidase activity measurements showed that, like *Sty*FrmR, *Ec*FrmR(P2A) and *Ec*FrmR(C35A) failed to respond to formaldehyde, confirming that Pro2 and Cys35 (W and X positions in the CsoR/RcnR family fingerprint; Fig. 1a) are essential for perception of formaldehyde (Fig. 4a). Replacement of His60 (position Y) resulted in high basal activity that was further enhanced in the presence of formaldehyde, whereas replacement of Thr64 (position Z) had no effect on the function of *Ec*FrmR (Fig. 4a). Additionally, by analogy to RcnR metal-sensing residues^17^, insertion of an Ala codon before Pro2 (*Ec*FrmR(A2*)) also resulted in formaldehyde insensitivity, albeit with higher basal P_*frm*_ activity, implicating the Pro2 imino group in formaldehyde sensing (Fig. 4a). Replacement of the only other cysteine residue (Cys70) in *Ec*FrmR did not impair the response to formaldehyde (Fig. 4a). The conclusion that Pro2 and Cys35 are required for *Ec*FrmR to respond to formaldehyde was supported by the formaldehyde-sensitivity of *E. coli* strains expressing *Ec*FrmR(P2A) and *Ec*FrmR(C35A) in place of *Ec*FrmR. Cultures expressing these variants exhibited enhanced sensitivity to formaldehyde, consistent with the low *frmRAB* expression observed in the reporter fusion experiments, presumably arising from constitutive repression *frmRAB* expression (Fig. 4a and b; Table 2).

### Reaction of *Ec*FrmR with formaldehyde

The modification of *Ec*FrmR by formaldehyde, and the competition with Zn(II), was analyzed by LC-MS. Without formaldehyde, the major species corresponded to the *Ec*FrmR monomer lacking the *N*-terminal methionine (10186.60 Da; predicted mass 10186.50 Da) with lower amounts of a disulfide-linked dimer (20371.02 Da; predicted unmodified dimer mass 20373.20 Da) (Table 2). After exposure to formaldehyde (8-fold molar excess) for 3 min the *Ec*FrmR monomer was still detected (10187.02 Da) along with new species of molecular mass 20396.87 Da, corresponding to an *Ec*FrmR dimer plus an additional mass of 23.67 Da, and 40768.73 Da, corresponding to a tetramer with an additional mass of 22.30 Da (Table 2; Fig. S3). The former modified species represents *Ec*FrmR dimers linked by two intermolecular methylene bridges (-CH_2_-) (net mass gain of 2 × 12 Da per *Ec*FrmR dimer); the latter modified species can be accounted for by an *Ec*FrmR tetramer in which each subunit participates in only one methylene bridge (total of two in the tetramer) and these cross-linked dimers are held together by one disulfide bond (Table 2; Fig. S3). Incubation with stoichiometric Zn(II) (4 Zn(II) per *Ec*FrmR tetramer), before or after formaldehyde treatment, yielded dimeric species of molecular masses 20396.99 Da and 20396.95 Da, respectively, indicating that Zn(II) ions did not prevent formaldehyde-dependent methylene bridge formation under these conditions.

Formaldehyde modification of *Ec*FrmR(P2A) and *Ec*FrmR(C35A) was examined because they failed to respond to formaldehyde *in vivo* (Fig. 4). Neither mutation affected the oligomeric state of the protein, as both variants eluted as tetramers upon size exclusion chromatography. LC-MS showed the presence of disulfide-linked *Ec*FrmR(P2A) dimers (20319.69 Da; predicted unmodified mass 20320.8 Da) in the absence or presence of formaldehyde (Table 2). However, exposure to formaldehyde without quenching permitted the detection of a monomeric *Ec*FrmR(P2A) species (10190.71 Da) with additional mass 29.91 Da, equivalent to an hydroxymethyl adduct. In contrast for *Ec*FrmR(C35A), a disulfide-linked dimer (20307.4 Da; predicted unmodified mass 20308.8 Da) that was not modified in the presence of formaldehyde was detected with or without quenching (Table 2). These data suggest that reaction with Cys35 is likely to be the first step in formaldehyde perception by *Ec*FrmR (Table 2). Taken together, the *in vivo* data and the LC-MS data are consistent with a mechanism in which *Ec*FrmR senses formaldehyde by the formation of methylene bridges between Pro2 and Cys35 residues of adjacent subunits; moreover, *Ec*FrmR is oxidized in air, forming dimers linked by intermolecular disulfide bonds.

### The structural response of *Ec*FrmR to formaldehyde modification and a mechanism for formaldehyde sensing

The crystal structure of formaldehyde-exposed *Ec*FrmR was determined to a resolution of 2.7 Å (protein databank identifier PDB: 5LBM). Like other members of the CsoR/RcnR family, *Ec*FrmR oligomerizes to form a disc-like tetramer, constructed from two homodimers, each of which forms one face of the disc. Each subunit consists of three helices (α1, residues 2–30; α2, residues 35–68; and α3, residues 73–91), linked by two short loops (L1, residues 31–34; and L2, residues 69–72), that are arranged as a flattened S-shape (Fig. 5a). The α3 helices slot together at the homodimer interface, such that each face of the tetramer is formed from a platform of five parallel helices with a hole at the centre (Fig. 5b). This arrangement differs from that seen in CsoR and *Sty*FrmR(E64H) (PDB: 5LCY) where the equivalent α3 helix is domain swapped onto the opposite face of the tetramer (Fig. S4)^23^,^24^. This difference between *Ec*FrmR and *Sty*FrmR(E64H) is striking especially as the conserved regions of the α1 and α2 helices of these proteins superpose well; superposition of the C_α_ atoms of residues 10–63 (α1-α2) of the uncross-linked or cross-linked chains of *Ec*FrmR with the uncross-linked *Sty*FrmR yielded root mean square deviation (RMSD) values of ~1.0 Å for both. However, superposition of the Cα atoms from the full length chains (either cross-linked or uncross-linked) gave RMSD values of ~5 Å, reflecting the different organisation of α3 within the tetramer, which could be a consequence of the differences in amino acid sequence in the region spanning the terminus of α2, L2 and the beginning of α3 (Fig. 1b).

The formaldehyde-exposed *Ec*FrmR tetramer is asymmetric. One face of the tetramer (A/B face) is comprised of an unmodified homodimer with electron density visible for residues Lys9-Lys91 of both polypeptides with a disordered *N*-terminal region (residues Pro2-Lys8). Electron density corresponding to Zn(II) or other metal ions was not detected; however the W-X-Y-Z fingerprint residues in the unmodified homo-dimer are located in similar positions in 3D space to those of the CsoR proteins from *Geobacillus thermodenitrificans* and *Thermus thermophilus* and some of these residues are likely to constitute the *Ec*FrmR Zn(II) binding site (Fig. S5)^24^,^25^. The XAS data supports the assignment of Cys35 and His60 as Zn(II) ligands (Fig. S1d). The residue at position Z (Thr64) does not interact with other residues of the W-X-Y-Z motif in either form of the *Ec*FrmR dimer, consistent with the lack of effect of the T64A mutation on formaldehyde-dependent de-repression of P_*frm*_ (Fig. 4a).

The homodimer forming the other face of the tetramer (A′/B′ face) is sandwiched against the first via a network of hydrophobic packing interactions between the secondary structural elements. In contrast to the A/B face, clear electron density is present for residues Pro2-Lys8 of both subunits on the A′/B′ face, resulting in an ordered extension to the *N*-terminal region of α1. The Pro2′-N atom is located within ~2.5 Å of the Cys35-SG atom of the corresponding subunit on the opposite face of the tetramer (Fig. 5b). There is extra density between these two atomic positions, indicating the presence of the formaldehyde cross-link, and a methylene bridge (-CH_2_-), as indicated by the LC-MS data, has been modeled at this location (Fig. 5c).

The crystal structure of *Ec*FrmR reveals both the free and signal-triggered states of the protein. One face of the *Ec*FrmR tetramer represents the conformation of the protein in the absence of formaldehyde (A/B face with a disordered Pro2), whilst the other face represents the conformation of the protein that has responded to formaldehyde and has formed the methylene bridge (A′/B′ face with an ordered Pro2) (Fig. 5b). ‘On’- and ‘off’-states of an RcnR/CsoR family member from a single source have not been observed previously and the structure presented here facilitates a detailed analysis of the conformational changes that occur upon reaction of *Ec*FrmR with formaldehyde to inhibit DNA-binding. Unlike *Ec*FrmR, the *N*-terminal region of *Sty*FrmR(E64H) is visible in the absence of the methylene bridge^23^. This showed that the *Sty*FrmR(E64H) *N*-terminal Pro2′ is located close to Cys35 (~3 Å between Pro2′-N and Cys35-SG atoms), poised to facilitate formaldehyde-specific cross-linking. With the exception of the domain swapped α3, the uncross-linked (A/B) face of *Ec*FrmR superposes well on the *Sty*FrmR structure (RSMD ~1.0 Å). This suggests that a remarkably subtle change in the locations of Pro2′ and Cys35 and the constraints imposed by the covalent methylene bridge cross-link contribute to creating a distinct difference in the relative conformation and orientation of the subunits in the cross-linked face compared to those of the uncross-linked face. Whilst the position of α3 is similar on both faces of the tetramer, there is a translational movement of α1 and α2 on the A′/B′ face, which slide across the equivalent helices on the opposite face by ~1.5 α-helical turns in response to formation of the methylene bridge. In addition, the *C*-terminal half of α2 twists and buckles towards α3, which alters the packing of these two helices, generating a 10° off-parallel angle between α3 and α2. This movement changes the overall size and shape of the A′/B′ face, which expands and elongates by ~10 Å on the diagonal in response to formaldehyde (Fig. 5b; Animation S1). Notably, expansion of the envelope of CsoR in response to Cu(I) was apparent in small angle X-ray scattering studies^27^. Furthermore, in addition to an H-bond network, involving His60-Tyr34-Glu80 that is thought to be involved in signal (Cu(I)) perception but not signal binding by *M. tuberculosis* and *G. thermodenitrificans* CsoR proteins, the *N*-terminal region of *G. thermodenitrificans* CsoR becomes ordered over the Cu(I)-binding site^24^. The stable ordering of the *N*-terminal regions of *Ec*FrmR and CsoR proteins upon signal perception establishes new interactions between the α1 and α2 helices of these proteins (Fig. S6). Thus, signal perception by *Ec*FrmR results in a H-bond interaction between Glu7 (α1) and Ser59 (α2), which is not present in the uncross-linked form of *Ec*FrmR, *Sty*FrmR(E64H) structure or in the Cu(I)-free form of *S. lividans* CsoR. The same two residue positions of Cu(I)-loaded *G. thermodenitrificans* CsoR (Glu22-Arg74) participate in an electrostatic interaction. Moreover, an equivalent interaction occurs in *M. tuberculosis* CsoR, but involving a slightly different residue position in α2 (Lys8-Glu63). Thus, it is suggested that *N*-terminal ordering and the establishment of interactions between α1 and α2 could be a common feature of signal perception and transduction in the CsoR/RcnR family that has only been revealed because both ‘on’ and ‘off’ states are captured in the *Ec*FrmR structure.

**Figure 2:**
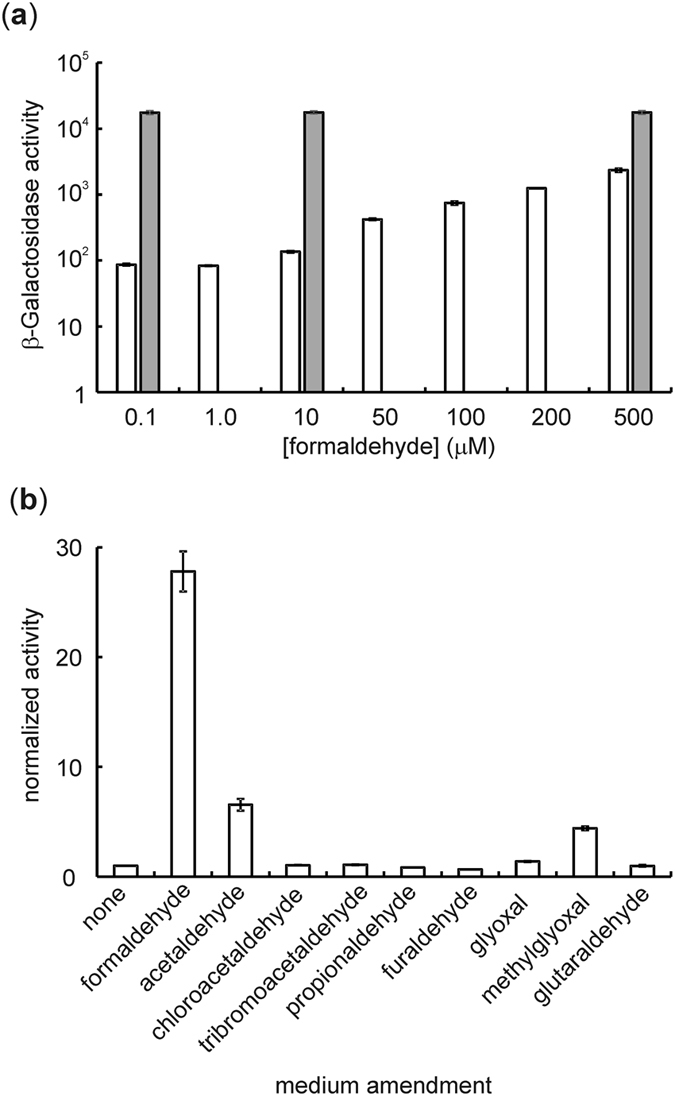
*Ec*FrmR-mediated repression of *frmRAB* expression is relieved by formaldehyde. (**a**) Cultures of *E. coli* PC677 carrying P_*frm*_-*frmR*-*lacZ* (open bars) or P_*frm*_-*frmR*_stop_-*lacZ* (gray bars) were grown as described in *Methods* in the absence and presence of the indicated concentrations of formaldehyde. β-Galactosidase activities (Miller units plotted on a log scale) were measured as a proxy for *in vivo* transcription from the *frmRAB* promoter. (**b**) β-Galactosidase activities (Miller units) of cultures of *E. coli* PC677 carrying P_*frm*_-*frmR*-*lacZ* were measured after anaerobic cultivation in the presence of the indicated aldehydes as described in *Methods*. Activities were normalized to that measured in the absence of formaldehyde. For both panels, the error bars represent the standard deviation from the mean (n = 3).

**Figure 3:**
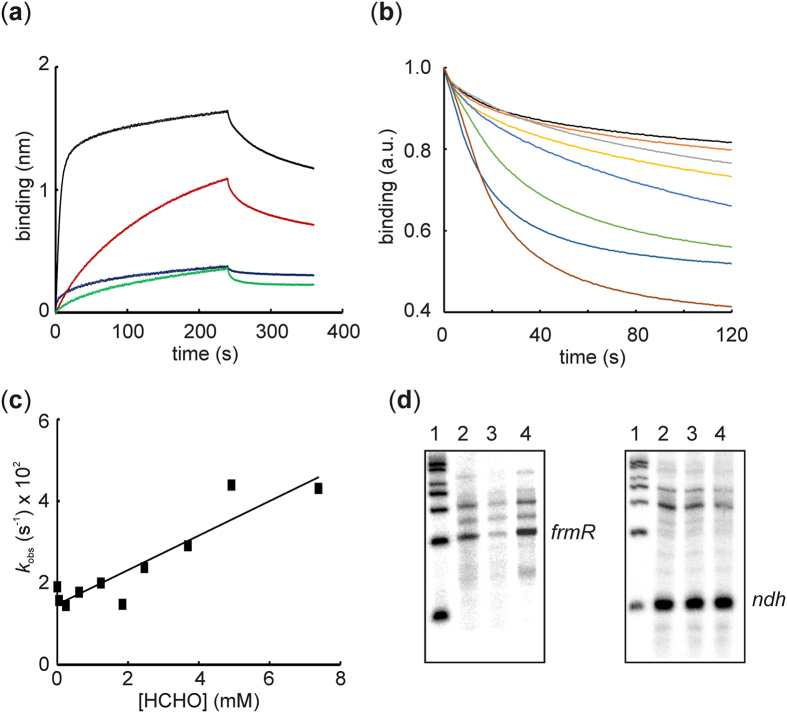
Formaldehyde enhances disassociation of the P_*frm*_-*Ec*FrmR complex. (**a**) Bio-Layer Interferometry (BLItz) assays. Reactions to evaluate the interaction of biotin-labeled P_*frm*_ DNA, immobilized on a streptavidin probe, with *Ec*FrmR were carried out with 10 different concentrations of *Ec*FrmR (Table S4A). Representative traces for *Ec*FrmR (6.16 μM tetramer, black line, 0.88 μM tetramer; red line), as well as *Ec*FrmR pre-treated with 200-fold molar excess of formaldehyde (0.88 μM tetramer; blue line), and *Ec*FrmR binding at a non-target DNA (P_*ydhY*_, 0.88 μM *Ec*FrmR tetramer; green line) are shown. (**b**) Pre-formed P_*frm*_-*Ec*FrmR complexes were exposed to 10 different concentrations (Table S4C) of formaldehyde and disassociation curves were recorded. Traces for 0 (black); 0.05 mM (orange); 0.25 mM (gray); 0.62 mM (yellow); 1.25 mM (blue); 3.69 mM (green); 4.92 mM (dark blue); 7.38 mM (brown) are shown. (**c**) Single exponential fits to formaldehyde disassociation curves were used to obtain the observed rate constants (*k*_obs_) which were plotted against formaldehyde concentration to obtain the apparent second order rate constant. (**d**) Inhibition of *frmRAB* transcription by *Ec*FrmR *in vitro* is relieved by formaldehyde. Reaction conditions are described in the *Methods* section. Left panel, P*frm*; right panel, P*ndh*. Lanes 1, RNA size markers, top to bottom: 600, 500, 400, 300, 200, 100 bases; Lanes 2, no *Ec*FrmR; lanes 3, 1 nM *Ec*FrmR tetramer; lane 4, 1 nM *Ec*FrmR tetramer pre-treated with 200-molar excess formaldehyde. The locations of the *frmR* and *ndh* are indicated.

**Figure 4:**
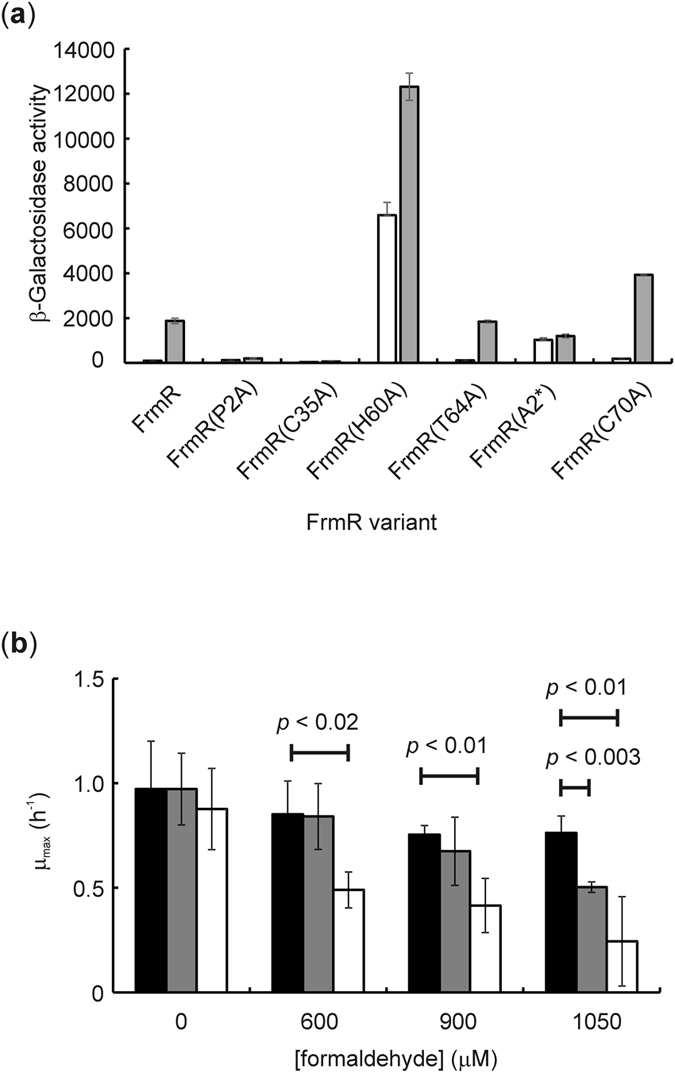
Identification of formaldehyde-insensitive *Ec*FrmR protein variants. (**a**) The P_*frm*_-*frmR*-*lacZ* reporter was modified to encode *Ec*FrmR variants with the indicated amino acid substitutions. Cultures of *E. coli* PC677 carrying these reporters were grown under anaerobic conditions in the absence (open bars) of presence (gray bars) of formaldehyde (250 μM) and β-galactosidase activities (Miller units) were measured as described in *Methods*. The error bars represent the standard deviation from the mean (n = 3). (**b**) Maximum growth rates (μ_max_) of *E. coli* MG1655 *frmRAB* mutant transformed with plasmids expressing the *frmRAB* operon from P_*frm*_ under the control of wild-type *Ec*FrmR (closed bars), *Ec*FrmR(P2A) (gray bars) or *Ec*FrmR(C35A) (open bars) cultured in the presence of the indicated initial concentrations of formaldehyde. The mean and standard deviations (n = 3) and the *p* values for one-tailed t-tests are shown.

**Figure 5:**
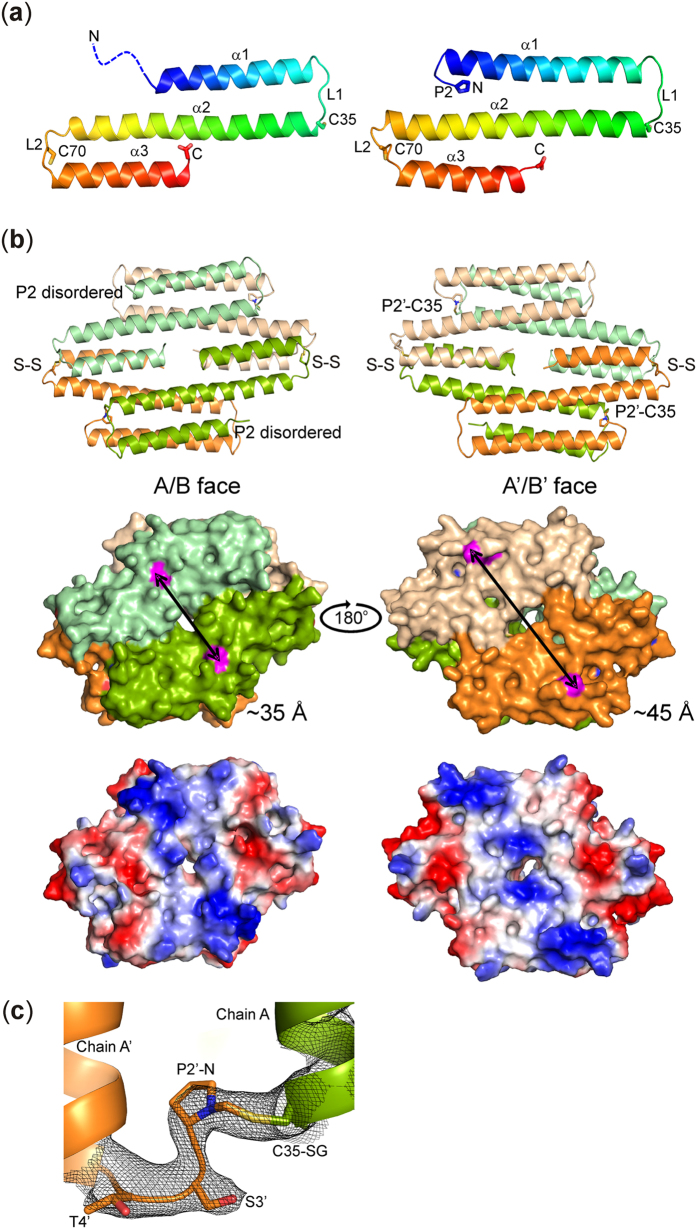
Structure of *Ec*FrmR. (**a**) Cartoon representations of uncross-linked (left) and cross-linked (right) *Ec*FrmR monomers colored blue (*N*-terminal) to red (*C*-terminal). Secondary structure elements (α-helices, α1 to α3; loops, L1 and L2) are labeled and the amino acid residues (single letter code, P2, C35 and C70) involved in cross-linking and disulfide bond formation are shown as sticks. The disordered *N*-terminal region in the uncross-linked subunit is represented by the blue dashed line. (**b**) A comparison of the overall size and shape of the uncross-linked (left) and cross-linked (right) faces of the *Ec*FrmR tetramer. The upper images show the arrangement of the helices on each face of the tetramer, the positions of the methylene bridges (P2′-C35) and the Cys70-Cys70′ disulfide bonds (S-S). The homodimer (A/B) on the uncross-linked face is drawn in shades of green and the cross-linked face (A′/B′) in shades of orange. The middle images show the expansion of the surface envelope upon cross-linking (black double headed arrow drawn between Arg14 Cα atoms, highlighted in pink). The lower images show the surface-charge on either side of the tetramer (red represents negative charge, blue positive charge and white neutral). (**c**) Section of the 2Fo-Fc map between chains A and A′ obtained when the coordinates for Pro2 and the methylene bridge were omitted from the refinement (black mesh, contoured at 1σ). Residues are indicated by their single letter codes.

The different conformational states of the two faces of formaldehyde-treated *Ec*FrmR have a dramatic effect on the pattern of surface charge and the position of protrusions on each face of the tetramer (Fig. 5b). There are two patches of positive charge either side of the central hole on both faces of the tetramer, which contain several residues implicated in DNA-binding (Arg14, Arg16, Gln41, Arg46 and Lys91)^24^,^30^,^31^. The differences in size and shape of the two faces of *Ec*FrmR in the crystal structure mean that these patches are separated by ~35 Å on the A/B face but by ~45 Å on the A′/B′ face. Whilst an asymmetric tetramer has been captured in the crystal structure, modeling suggests that both faces of *Ec*FrmR could adopt the compact DNA-binding conformation in the absence of formaldehyde-induced cross-linking. However, models of a fully cross-linked tetramer, in which subunits A and A′ and B and B′ were both linked by two methylene bridges, as implied by the LC-MS data, contained many inter-subunit clashes, indicating that both faces of *Ec*FrmR might not be able to adopt the extended conformation whilst retaining the tetrameric state. The presence of only two methylene bridges in the structure of the *Ec*FrmR tetramer raises the possibility that, at least under some conditions, the formation of the third and fourth cross-links might be subject to negative cooperativity, as observed for binding the third and fourth Zn(II) ions to *Ec*FrmR (see above) and that the fully cross-linked protein might disassociate into dimers incapable of DNA-binding. The negative cooperativity is also consistent with the effects of the H60A mutation on P_*frm*_-*lacZ* activity (Fig. 4a). This mutant shows high constitutive promoter activity, similar to the stop codon mutant (Fig. 2a). However, the maximum induction by formaldehyde is substantially lower. This contrasts with the ability of Ni(II) and Co(II) repress P_*rcn*_ to the same extent as a stop codon mutant of RcnR^16^. The His60 residue of *Ec*FrmR is within H-bond distance of Cys35 in the unmodified dimer (3.2 Å), and makes no obvious interactions with side chain or main chain residues in the methylene-bridged structure. Hence, the H60A mutation may mimic the modified form of the protein at all four sites, instead of two, resulting in greater de-repression.

**Figure 6:**
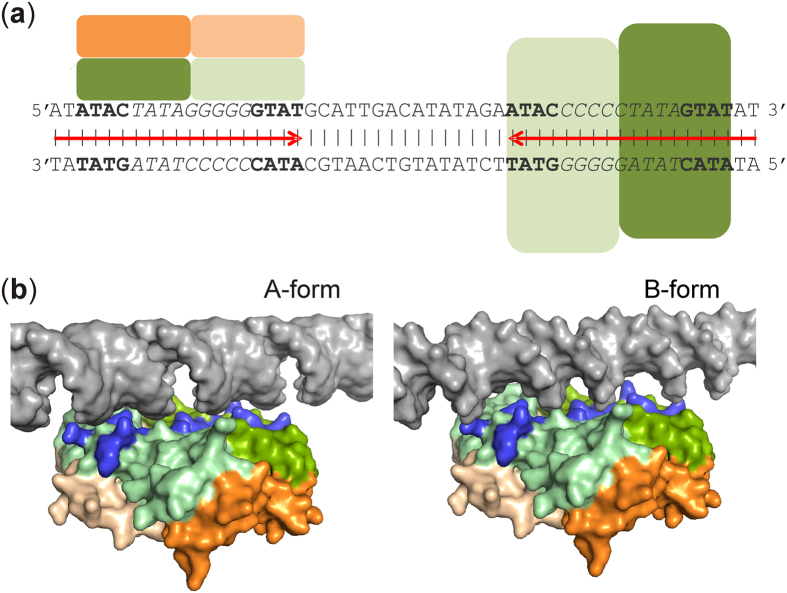
Modeling the P_*frm*_–*Ec*FrmR complex. (**a**) The DNA sequence of the *frmRAB* promoter region (P_*frm*_) contains tandem *Ec*FrmR binding sites consisting of ATAC/GTAT inverted repeats (bold) separated by G/C-rich tracts (italic) that form a larger inverted repeat (convergent red arrows). The size of *Ec*FrmR (subunits colored in shades of green and orange as in Fig. 5b) suggests that two tetramers could bind to the *frmRAB* promoter region. One *Ec*FrmR tetramer (side view) is shown on the top face of the DNA sequence and the other (top view) behind the DNA sequence, offset by approximately a quarter turn relative to the first tetramer. (**b**) Models of binary complexes formed from *Ec*FrmR and A- or B-form DNA. One of the tandem *Ec*FrmR binding sites of P_*frm*_ (dark gray) is modeled as A- (left) and B-form (right) DNA. *Ec*FrmR is shown as surface representation with subunits colored in shades of green (uncross-linked A/B face) and orange (cross-linked A′/B′ face), with the amino acid side-chains on the A/B face that are implicated in DNA-binding highlighted in blue.

It has been suggested that CsoR/RcnR family members recognize their DNA target by a combination of: (i) shape selectivity, resulting from the propensity of the central GC tract to adopt the A-DNA form; (ii) specific interactions with the flanking inverted repeats; and (iii) non-specific binding to distant DNA that might result in DNA-wrapping^30^,^31^. Operator sequences for CsoR/RcnR family members have been classified into two groups: type I sites consist of GC tracts (3–8 bp) flanked by AT-rich inverted repeats; type II sites have shorter interrupted GC tracts^30^,^31^. The *E. coli* P_*frm*_ contains a large inverted repeat centered at −29 relative to the predicted transcriptional start site (Fig. 6a). This region contains tandem type I FrmR-binding sites consisting of 9 bp GC-rich tracts flanked by ATAC/GTAT inverted repeats (Fig. 6a). The *E. coli rcnR-A* intergenic region also possesses tandem type I RcnR-binding sites containing a TACTGGGGGGNAGTA motif, which imparts some A-form DNA character on this region of DNA, and one RcnR tetramer binds at each site on the same face of the DNA helix^31^. The recently reported structure of the site-directed mutant *Sty*FrmR(E64H), which responds to Co(II), Zn(II) and formaldehyde *in vivo* showed the presence of positively-charged surface patches for protein that had not been exposed to formaldehyde^22^,^23^. As noted above for the uncross-linked surface of *Ec*FrmR (Fig. 5b), the *Sty*FrmR(E64H) positively-charged patches were also separated by ~35 Å (distance between the Arg14 C_α_ atoms)^23^. These data were used to inform models of the P*frm*–*Ec*FrmR complexes with A- and B-form DNA (Fig. 6). Models with both A- and B-form DNA suggested that residues (Lys10, Arg14, Arg16 and Arg17) forming the two positively-charged protrusions on the A/B face could interact with the major grooves of the DNA, with Lys91 from the central hole packing into the minor groove, but the better fit is with B-form DNA. It is noted that in the bacterial cell P_*frm*_ DNA is unlikely to be exclusively in A- or B-form, but more likely a hybrid that will be further distorted upon binding of the FrmR tetramer. Nevertheless, the ~45 Å that separates the positively-charged patches on the cross-linked A′/B′ face precludes convincing interactions with either A- or B-form DNA. These differences suggest a mechanism for de-repression of the *frmRAB* promoter in response to formaldehyde. In the DNA bound state, Lys91 is held in a surface-exposed position by an ion pair interaction between Arg14 and the *C*-terminal carboxyl. Upon cross-linking, α1 and α2 are pulled away from the centre of the face of the tetramer, with loop 2 acting as a pivot point. This motion pulls Arg14 away from Lys91, breaking the interaction with the *C*-terminal carboxyl, which causes it to rotate by ~90° into the central hole, forming a new interaction with the guanidyl group of Arg46. The surface-exposed side-chain of Lys91 moves into the hole, burying the NZ atom so that it is no longer available to interact with DNA (Fig. S7). In addition, the motion of α1 and α2 increases the distance between the cluster of residues within the positively-charged protrusions by ~10 Å, such that Lys10, Arg14, Arg16 and Arg17 can no longer interact with the major groove, thus breaking the complex between *Ec*FrmR and DNA. The centers of the tandem binding sites at P_*frm*_ are separated by 31 bp compared to 19 bp for the *rcnR-A* intergenic region, raising the possibility that, unlike RcnR, both faces of a single *Ec*FrmR tetramer could participate in binding to the tandem sites at P_*frm*_.

### Concluding remarks

*Ec*FrmR is the first example of a CsoR/RcnR family protein where the asymmetry of the tetramer in the crystal structure reveals the conformational changes induced by signal perception that lead to de-repression of target promoters. *In vivo* and *in vitro* experimental evidence show that the *E*cFrmR specifically reacts with the toxic chemical formaldehyde, resulting in the formation of inter-molecular methylene bridges between adjacent Pro2 and Cys35 residues. In the absence of formaldehyde the *Sty*FrmR(E64H) structure indicates that the *N*-terminal regions are ordered, such that the *N*-terminal Pro residues are in close proximity to Cys35 of an adjacent subunit^23^. The conservation of His60 and its proximity to Cys35 in the adjacent subunit suggests that it could act to abstract a proton from the thiol group to facilitate the initial reaction with formaldehyde forming an *S*-hydroxymethyl adduct (LC-MS data Table 2). The initial hydroxymethylated Cys35 residues appear to be associated with one surface of the *Ec*FrmR disc permiting nucleophilic attack by Pro2 residues associated with the opposite face of the *Ec*FrmR disc resulting in methylene bridge formation (detected as the oxidized *Ec*FrmR tetramer with additional mass 22.3 Da in LC-MS; Table 2, Fig. S3); an example of an *N*-terminal Pro residue acting as a nucleophile has been reported for the DNA-repair enzyme, formamidopyrimidine-DNA glycosylase^32^. The covalent capture of the Pro2 residues of one face of *Ec*FrmR could contribute to the disordering of the uncross-linked *N*-terminal regions, which is apparent in the crystal structure reported here (Fig. 5). Alternatively, fully uncross-linked *Ec*FrmR and *Sty*FrmR could differ in the degree of flexibility in their *N*-terminal regions despite the high level of sequence conservation (Fig. 1b). The *N*-terminal disordering captured in the formaldehyde-treated *Ec*FrmR crystal structure could account for the negative cooperativity observed for Zn(II) binding and perhaps in formaldehyde reactivity. Formaldehyde-induced methylene bridge formation (two per tetramer) expands the A′/B′ surface of *Ec*FrmR resulting in a similar change in size to that observed for *G. thermodenitrificans* CsoR upon Cu(I) binding^24^. Interestingly, CsoR does not use direct coordination of the signal molecule via the *N*-terminus, unlike FrmR and RcnR. An allosteric network has been proposed for CsoR that involves an interaction between His60 and two other residues (Tyr34 and Glu80, numbered based on the *N*-terminus of FrmR and RcnR)^33^. However, these interactions are not present in FrmR and RcnR, suggesting that the allosteric network may initiate at the *N*-terminus upon signal perception. This notion is consistent with the observation that the *G. thermodenitrificans* CsoR structure shows the *N*-terminus capping the Cu(I)-binding site, but not directly coordinating the Cu(I) atom^24^. Thus, despite specific differences between RcnR, FrmR, and CsoR in the connectivity between signal perception residue(s) and the common DNA-binding interface of the proteins, an ordering of the *N*-terminal region and α1 may be a common feature of signal perception in this protein family. The inability of Zn(II) to drive *Ec*FrmR off DNA is likely to be because Zn(II) binding cannot order the *N*-terminal region of α1. Ultimately, all four *Ec*FrmR *N*-terminal Pro2 residues can be methylene bridged to their partner Cys35 residues resulting in derepression of *frmRAB* expression (detected as *Ec*FrmR dimers +24 Da in LC-MS; Table 2; Fig. S3).

Finally, it is notable that the second *Ec*FrmR Cys residue (Cys70), which is not conserved in *Sty*FrmR, can form a disulfide bond linking loops 2 of the A/B′ and the B/A′ chains. The effect of this disulfide on the ability of all four *Ec*FrmR subunits to undergo formaldehyde modification to fine-tune the *Ec*FrmR response has not been assessed here, but the potential for *Ec*FrmR to act as a redox sensor, through the formation of a disulfide bond, is of interest because formaldehyde exposure is associated with glutathione-depletion and oxidative stress in higher organisms^34^,^35^,^36^,^37^,^38^. Work is on-going to determine the possible role of glutathione (*S*-hydroxymethylglutathione is formed in *E. coli* exposed to formaldehyde) and oxidative stress (disulfide bond formation) in regulating *Ec*FrmR activity *in vivo* as well as to determine the structure of the P_*frm*_-*Ec*FrmRcomplex.

## Methods

### Bacterial strains, plasmids and oligonucleotides

These are listed in Supplementary Table S1. The *P*_*frm*_-*frmR*-*lacZ* plasmid was constructed from pPC163, which contains the *P*_*nik*_ promoter and *lacZ* ligated into pACYC184^17^. A fragment consisting of 499 bp of DNA located upstream of *frmA*, which included *frmR,* was ligated into the *Eag*I and *Sal*I sites of pPC163, replacing the *P*_*nik*_ cassette. For overproduction of *Ec*FrmR, the *frmR* gene was amplified from *E. coli* K12 genomic DNA with primers JI174 and JI175, and ligated into pET22b at the *Nde*I and *Nco*I sites. Mutations in *frmR* were introduced by overlap PCR or by the Quikchange protocol (Stratagene) using the appropriate oligonucleotide primers (Table S1). Formaldehyde-sensitivities of *E. coli* JRG6703, harboring either pGS2497 (*frmR* wild-type), pGS2547 (*frmR* P2A) or pGS2548 (*frmR* C35A) were determined using aerobic, Luria-Bertani (LB) medium (200 μl), 96-well plate cultures. Formaldehyde (0–1650 μM) was added and the cultures were grown at 37 °C with shaking (250 rpm). Growth was monitored using a Sunrise absorbance reader (Tecan) at A_595_ for 500 min at intervals of 20 min. All cultures were grown in triplicate. The maximum growth rate (μ_max_ h^−1^) for each strain was calculated.

### Isolation of *Ec*FrmR and selenomethionine (Se-Met) incorporated *Ec*FrmR protein

For isolation of *Ec*FrmR and the P2A and C35A variants, cultures of the *E. coli* expression strains (JRG6782, 6783 and 6784) were grown at 37 °C in LB medium containing ampicillin (100 μg ml^−1^), to an OD of ~0.6, at which point 1 mM IPTG was added and the cultures were incubated for a further 3 h. To obtain selenomethionine (Se-Met) incorporated *Ec*FrmR, cultures were grown to OD ~0.6 in LB, then pelleted and resuspended into Se-Met over-expression medium (60 mM K_2_HPO_4_, 8 mM (NH_4_)_2_SO_4_, 33 mM KH_2_PO_4_, 2 mM tri-sodium citrate, 54 mM glycerol, 4 mM adenine, 2 mM guanosine, 4 mM thymine, 4 mM uracil, 4 mM MgSO_4_, 12 mM thiamine, L-lysine (100 mg l^−1^), L-phenylalanine (100 mg l^−1^), L-threonine (100 mg l^−1^) L-isoleucine (50 mg l^−1^), L-leucine (50 mg l^−1^), L-valine (50 mg l^−1^) and seleno –L –methionine (40 mg l^−1^)) before further incubation for 1 h at 37°C prior to induction of *Ec*FrmR protein expression with IPTG. Cells were lysed after re-suspension in Buffer A (50 mM Tris, 0.1 M NaCl; pH 8.0) by sonication (Soniprep150 ultrasonic disintegrator) at ~16 microns for 2 cycles of 20 s. The lysate was cleared by centrifugation (10 min, 70000 *g*) and the resulting cell-free extract was applied to a Heparin-HP column (GE Healthcare) and *Ec*FrmR was eluted using a NaCl gradient (0 to 0.5 M in 50 ml) in Buffer A. Fractions containing *Ec*FrmR protein were combined and dialyzed overnight against 50 mM Tris-HCl buffer, pH 8.0 at 4 °C. Micro-crystals of *Ec*FrmR were collected by centrifugation and dissolved in 1 ml of 1 M NaCl in buffer A (see above). The sample was then applied to a HiLoad Superdex 200 column (GE Healthcare) equilibrated with 50 mM Tris-HCl, 0.5 M NaCl (pH 8.0). *Ec*FrmR-containing fractions were pooled and the purity of the samples was assessed by SDS-polyacrylamide gel electrophoresis. For crystallization, protein was concentrated to 10 mg ml^−1^ using a VivaSpin device with MWCO 30000 (GE Healthcare). The authenticity of *Ec*FrmR was confirmed by total amino acid analysis (Alta Biosciences; University of Birmingham, UK) allowing calibration of *Ec*FrmR protein concentrations measured by either the Bio-Rad protein reagent or using the calculated molar extinction coefficient for *Ec*FrmR^39^,^40^.

### Zn(II) binding Assays

Zn(II) titrations, XAS and EXAFS analyses are described in Supplementary material.

### Analytical ultracentrifugation

Details are provided in the supplementary material.

### Estimation of the number of reactive thiols in *Ec*FrmR

Reactive protein thiols were estimated by the method of Thelander^41^. In a Quartz cuvette, a total reaction volume of 1 ml was prepared consisting of purified protein (100 μl) diluted with protein elution buffer (50 mM Tris, 0.5 M NaCl (pH 8.0) and 200 μl DTNB solution (0.2 ml 0.4% DTNB in ethanol and 1.8 ml 1 M Tris-HCl, pH 8.0). Absorbance at 412 nm was measured and the 2-nitrothiobenzoate concentration was used to calculate the number of reactive sulfydryl groups.

### Mass spectrometry

Protein samples (~12 μM) for LC-MS were incubated with either an 8-fold or 40-fold molar excess of formaldehyde to *Ec*FrmR tetramer concentration at room temperature. After 3 min, reactions were quenched with 10 mM glycine. Samples were loaded onto an Agilent 1260 Infinity liquid chromatograph fitted with an Agilent Extended C18 column (2.1 mm × 50 mm) and eluted with a gradient of 5-95% acetonitrile in 0.1% formic acid at 400 μl min^−1^ over 8 min. The eluate was directly coupled to an Agilent 6530 Q-ToF mass spectrometer fitted with an electrospray ionisation (ESI) source for determination of the masses of species detected in the chromatograph.

For ICP-MS, *Ec*FrmR (200 μM) in 50 mM Tris (pH 8.0) buffer containing 0.5 M NaCl or buffer was incubated with concentrated nitric acid (1:1 ratio) at 60 °C for 1 h. Samples were cooled, diluted with dH_2_O (up to 10 ml) and filtered before analysis on a Perkin Elmer Nexlon ICP-MS system. Ions were quantified using a dilution series of certified multi-element reference standard (Sigma-Aldrich). Counts per second values for elements in the buffer and protein samples were then compared with the calibration curve to determine actual concentrations.

### Bio-layer interferometry (BLItz)

Biotinylated-promoter DNA for *frm* (P_*frm*_) and *ydhY* (P_*ydhY*_) was amplified from *E. coli* MG1655 genomic DNA by PCR using appropriate oligonucleotide primers (Table S1). Where indicated, *Ec*FrmR tetramers were incubated with formaldehyde (up to an 800-fold molar excess) for 3 min (before quenching with 10 mM glycine) or Zn(II) (16-fold molar excess) at room temperature, prior to measurements. Analysis of the interaction between purified *Ec*FrmR and biotinylated-promoter DNA was carried out using the BLItz system (FortéBio), at 20 °C. Streptavidin (SA) biosensors (FortéBio) were hydrated by soaking the tips in 250 μl protein elution buffer (50 mM Tris-HCl, pH 8.0 containing 0.5 M NaCl) for at least 10 min. The measurements were made using the Advanced Kinetics function of the BLItz Pro 1.1.0.31 software (FortéBio). The sequence for each run was as follows: (1) baseline step with protein elution buffer for 60 s, (2) loading step with 100 nM biotinylated DNA in buffer for 240 s, (3) baseline step with buffer for 60 s, (4) association step with various concentrations of purified *Ec*FrmR in buffer for 240 s, (5) dissociation step with buffer (or formaldehyde-containing buffer) for 120 s. Baseline and dissociation steps were carried out by placing the biosensor in a black 500 μl Eppendorf tube filled with 250 μl buffer. Loading and association steps were carried out by placing the biosensor in the drop holder containing either 5 μl of P_*frm*_ DNA or *Ec*FrmR protein depending on the step. For each run a new biosensor was used. The BLItz Pro 1.1.0.31 software using global fitting and correcting both association and dissociation curves was used for analysis.

### β-Galactosidase reporter experiments

Starter cultures of *E. coli* PC677 (Δ*frmR*) containing the appropriate reporter plasmid (wild type or mutant) were grown aerobically in LB medium with chloramphenicol (0.1 mM) to an OD_600_ ~0.5. These cultures were then used to inoculate 1.87 ml of LB medium with 0.1 mM chloramphenicol in capped microcentrifuge tubes in triplicate with aldehyde compounds as indicated. Cultures were grown overnight (12–15 h) before measurement of β-galactosidase activity^42^. The maximal aldehyde concentrations that inhibited growth by <15% were: 700 μM formaldehyde, 7 mM acetaldehyde, 100 μM chloroacetaldehyde, 11 μM tribromoacetaldehyde, 4 mM propionaldehyde, 10 mM furaldehyde, 3.5 mM glyoxal, 350 μM methylglyoxal and 500 μM glutaraldehyde.

### *In vitro* transcription reactions

The promoter and part of the coding regions of *frmR* and *ndh* (~200 bp upstream of the start codon to ~190 bp (*frmR*) or ~100 bp (*ndh*) into the gene) were amplified from *E. coli* MG1655 genomic DNA using appropriate oligonucleotides (Table S1). These DNA fragments (~0.1 pmol) were incubated for 30 min at 37 °C in a 10.5 μl reaction volume containing 40 mM Tris-HCl, pH 8.0, 10 mM MgCl_2_, 1 mM dithiothreitol, 75 mM KCl, 0.1 mM EDTA, 5% glycerol, 250 μg ml^−1^ bovine serum albumin, 20 units of RiboLoc RNase inhibitor (Fermentas), 1 pmol *E. coli* RNA polymerase holoenzyme (New England BioLabs, Inc.) and 0 or 1 nM *Ec*FrmR tetramer. *Ec*FrmR was reduced with 10 mM dithiothreitol and when required treated with 200-fold molar excess of formaldehyde for 5 min at room temperature, before quenching with 10 mM glycine. Transcription was initiated by the addition of 2 μl solution containing UTP at 50 μM; ATP, CTP and GTP at 1 mM; and 2.5 μCi of [α-^32^P]UTP (800 Ci mmol^−1^; PerkinElmer Life Sciences), followed by incubation for 15 min at 37 °C. Reactions were terminated by the addition of 12.5 μl Stop/Loading dye solution (95% formamide, 20 mM EDTA, pH 8, 0.05% bromophenol blue, 0.05% xylene cyanol). Samples (10 μl) of each reaction were loaded onto a 6% acrylamide, 1x TBE, 8 M urea gel and analysed using a phosphorimager (Typhoon 700; GE Healthcare). Markers (0.1–1 kb) were prepared using Perfect RNA Marker template mix (Novagen). A 20 μl reaction containing 0.75 μg of RNA template mix, 80 mM HEPES, pH 7.5, 12 mM MgCl_2_, 10 mM NaCl, 10 mM dithiothreitol, 2 mM ATP, 2 mM GTP, 2 mM CTP, 0.1 mM UTP, 5 μCi of [α-^32^P]UTP (800 Ci mmol^−1^, PerkinElmer Life Sciences), 20 units of RiboLoc RNase inhibitor (Fermentas) and 50 units of T7 RNA polymerase (Novagen), was incubated for 1 h at 37 °C, before storing at −20 °C. Markers from ~20 ng template were used for gel calibration.

### Crystallization and structural determination of *Ec*FrmR

Crystals of SeMet-labeled *Ec*FrmR (in 50 mM Hepes pH 7.5 and 0.5 M NaCl) were grown in 0.2 M MgCl_2_, 0.1 M Na cacodylate pH 6.5 and 31% PEG 2000. Crystals were harvested and cryoprotected in their mother liquor with an additional 25% ethylene glycol, before a single-wavelength (λ 0.9798 Å) anomalous dispersion (SAD) experiment (100 K) was carried out on beamline i03 at the Diamond Light Source (Table S3). The data were auto-processed using Xia2^43^ (XDS/Aimless) in space group P3_1_12 and AutoSharp^44^ was used to locate eight selenium sites (four full occupancy and four half occupancy) and build the basic polypeptide structure within the asymmetric unit, before multiple rounds of structure building and refinement using COOT^45^, Buccaneer^46^ and Refmac5^47^ from CCP4i^48^ were carried out to produce a poly-alanine model. The six subunits in the asymmetric unit comprised three half tetramers that were related by translational NCS, with poor electron density for one dimer. Subsequent higher resolution data (2.7 Å) were collected on beamline i03 (100 K) from a second SeMet-labeled crystal (λ = 0.9763 Å) that had been soaked in formaldehyde for 3 h before cryo-cooling. These data were in spacegroup P3_1_, with a tetramer of *Ec*FrmR in the asymmetric unit (Table S3). The poly-alanine model was used in molecular replacement^48^ as a starting point to build and refine the full structure of *Ec*FrmR using COOT^45^, ArpWarp^49^and Refmac5^47^ The final structure was refined to R_factor_/R_free_ values of 0.21 and 0.27, respectively, and was validated using Molprobity^50^. Structure superpositions were made using the SuperPose software^51^.

## Additional Information

**Accession code**: Structural data have been deposited in with the PDB; accession code 5LBM.

**How to cite this article:** Denby, K. J. *et al*. The mechanism of a formaldehyde-sensing transcriptional regulator. *Sci. Rep.* **6**, 38879; doi: 10.1038/srep38879 (2016).

**Publisher's note:** Springer Nature remains neutral with regard to jurisdictional claims in published maps and institutional affiliations.

## Supplementary Material

Animation File 1

Supplementary Materials

Supplementary Table S4

## Figures and Tables

**Table 1:** Rate constants for *Ec*FrmR DNA interactions.

Reaction^a^	*k*_f_ (M^−1^ s^−1^)^b^	*k*_r_ (s^−1^)^a^	K_d_ (nM)	*k*_app_ (M^−1^ s^−1^)
*Ec*FrmR + P_*frm*_ ↔ P_*frm*_ − *Ec*FrmR	13000 ± 390	0.0028 ± 0.000086	220	—
*Ec*FrmR − (Zn)_4_ + P_*frm*_ ↔ P_*frm*_ − *Ec*FrmR − (Zn)_4_	5660 ± 165	0.003 ± 0.000078	520	—
P_*frm*_ − *Ec*FrmR + HCHO → *Ec*FrmR − HCHO + P_*frm*_	—	—	—	4.2
P_*frm*_ − *Ec*FrmR − (Zn)_4_ + HCHO → *Ec*FrmR − (Zn)_4_ − HCHO + P_*frm*_	—	—	—	0.7
*Ec*FrmR + P_*ydhY*_ ↔ P_*ydhY*_ − *Ec*FrmR	1950 ± 1000	0.007 ± 0.00017	3600	—

^a^The BLItz data used to calculate the kinetic parameters shown are provided in Table S4. The data were fitted to a 1:1 binding model to derive *k*_f_, *k*_r_ and K_d_ values using all the sample data simultaneously (Global fitting).

^b^Value and standard error.

**Table 2:** Liquid chromatography mass spectrometry analyses of *Ec*FrmR proteins treated with formaldehyde.

Protein sample	Measured mass (Da)	Relative abundance (a. u.)	Mass difference(Da)^a^	Comment^b^
*Ec*FrmR	10186.60	2.0 × 10^7^	0.1	FrmR monomer
	20371.02	4.4 × 10^6^	−2.18	FrmR disulfide-linked dimer
HCHO-treated *Ec*FrmR	10187.02	1.8 × 10^6^	0.52	FrmR monomer
	20396.87	2.0 × 10^5^	23.67	FrmR dimer with 2 methylene bridges
	40768.73	2.0 × 10^5^	22.30	Two FrmR dimers, each with 1 methylene bridge, linked to form a tetramer by 1 disulfide bond
HCHO-treated *Ec*FrmR plus Zn(II)	10187.01	1.8 × 10^6^	0.51	FrmR monomer
	20396.95	3.0 × 10^5^	23.75	FrmR dimer with 2 methylene bridges
Zn(II)-treated *Ec*FrmR plus HCHO	10187.04	1.8 × 10^6^	0.54	FrmR monomer
	20396.99	4.0 × 10^5^	23.79	FrmR dimer with 2 methylene bridges
HCHO-treated *Ec*FrmR(P2A)	20319.69	4.0 × 10^5^	−1.11	FrmR(P2A) disulfide-linked dimer
HCHO-treated *Ec*FrmR(P2A)^c^	10190.71	6.8 × 10^5^	29.91	Hydroxymethylated FrmR(P2A) monomer
HCHO-treated *Ec*FrmR(C35A)	20307.40	2.8 × 10^6^	−1.40	FrmR(C35A) disulfide-linked dimer
HCHO-treated *Ec*FrmR(C35A)^c^	20307.21	1.7 × 10^5^	−1.59	FrmR(C35A) disulfide-linked dimer

^a^Difference between predicted mass and measured mass.

^b^Interpretation of mass difference; disulfide bond, −2.2 Da; methylene bridge, net mass gain 12 Da; hydroxymethylation, net mass gain 30 Da.

^c^Exposure to formaldehyde without quenching.
