# Supplementary material for: The mechanism of a formaldehyde-sensing transcriptional regulator
Source: Sci Rep. 2016 Dec 9;6:38879. doi: 10.1038/srep38879 (PMC5146963; doi:10.1038/srep38879)
Supplement: Supplementary Materials [file srep38879-s2.pdf]

Supplementary materials

The mechanism of a formaldehyde-sensing transcriptional regulator

Katie J. Denby[1,*], Jeffrey Iwig[2,*], Claudine Bisson[1,*], Jodie Westwood[1], Matthew D. Rolfe[1], Svetlana E. Sedelnikova[1], Khadine Higgins[3,4], Michael J. Maroney[3], Patrick J. Baker[1], Peter T. Chivers[2,5] and Jeffrey Green[1]

[1]Department of Molecular Biology and Biotechnology, University of Sheffield, Sheffield, S10 2TN, UK. [2]Department of Biochemistry and Molecular Biophysics, Washington University School of Medicine in St. Louis, St. Louis, MO, 63110, USA; present address: Carmot Therapeutics, Inc. San Francisco, CA 94158, USA. [3]Department of Chemistry, University of Massachusetts-Amherst, Amherst, MA 01003, USA. [4]present address: Department of Chemistry, Salve Regina University, Newport, Rhode Island 02840, USA. [5]Departments of Biosciences and Chemistry, Durham University, Durham, DH1 3LE, UK. *These authors contributed equally to this work. Correspondence and requests for materials should be addressed to P.J.B. (email: p.baker@sheffield.ac.uk), P.T.C. (email: peter.chivers@durham.ac.uk) or J.G. (email: jeff.green@sheffield.ac.uk).

Contents

Supplementary methods

Zn(II)- binding assays

X -ray Absorption Spectroscopy (XAS)

Analytical ultracentrifugation

Supplementary Tables

Table S1. Bacterial strains, plasmids and oligonucleotides

Table S2. Selected EXAFS fits for the Zn(II) FrmR complexes

Table S3. Crystallographic data

Supplementary Figures

Figure S1. The *E. coli* FrmR tetramer binds Zn(II).

Figure S2. Zn(II)-loaded FrmR exhibits an impaired response to formaldehyde.

Figure S3. Schematic of possible species of *Ec*FrmR formed by oxidation and reaction with formaldehyde.

Figure S4. Section of the *Ec*FrmR structure showing the connectivity between α2 and α3.

Figure S5. Superposition of the Cu(I)-binding site of CsoR and the putative Zn(II)-binding site of FrmR.

Figure S6. Signal-dependent interactions between $\alpha1$ and $\alpha2$ of three members of the CsoR/RcnR family.

Figure S7. Comparison of the conformation of the side-chains around the central hole of FrmR.

Animation file

An animation showing the conformational changes that occur when formaldehyde-induced methylene bridges are formed on one face (orange and beige subunits) of the *Ec*FrmR tetramer.

References

Supplementary Methods

Zn(II) binding assays. In a fluorescence cuvette (1 ml), *Ec*FrmR protein was diluted in protein elution buffer (50 mM Tris-HCl, pH 8.0 containing 0.5 M NaCl) to give a peak fluorescence intensity of at least 100 a.u. on a Cary Eclipse Fluorescence Spectrophotometer. Protein samples were maintained at 25°C for emission spectra (290-400 nm; excitation at 278 nm). FrmR was titrated with increasing zinc and manganese concentrations, typically to a 10-fold molar excess of [Zn(II)/Mn(II)]:[tetrameric *Ec*FrmR]. The peak intensity of the fluorescence emission at 304 nm was measured and the value was corrected to account for dilution of the protein.

Zn(II) binding affinity experiments were conducted in 10 mM Hepes (pH 7.5), 150 mM NaCl, 5% glycerol, with *Ec*FrmR being twice desalted in the same buffer with Microbiospin 6 columns (Bio-Rad) to remove DTT and EDTA. Separate samples contained 500 nM mag-fura 2 and the indicated Zn(II) concentrations with or without *Ec*FrmR. Samples were allowed to equilibrate 3 h before scanning. Fluorescence measurements were obtained on a Cary Eclipse fluorescence spectrophotometer in a 150 μl Hellma quartz cuvette (slit widths, 5 nm; $\lambda_{excitation}$, 320 nm; and $\lambda_{emission}$, 440-560 nm). Titrations were repeated three times.

X-ray Absorption Spectroscopy (XAS). Protein samples were syringed into polycarbonate XAS holders that were wrapped in kapton tape, and rapidly frozen in liquid nitrogen and XAS data were collected as previously described on beamline 9-3 at Stanford Synchrotron Radiation Laboratory (SSRL) in buffers containing NaBr or NaCl[1]. These data were collected at 10 K using a liquid helium cryostat (Oxford Instruments). The ring conditions were 3 GeV and 80-100 mA. Beam line optics consisted of a Si(220) double-crystal monochromator and two rhodium coated mirrors, a flat mirror before the monochromator (M_0) for harmonic rejection and vertical columnation, and a second bent, cylindrical mirror after the monochromator for focusing (M_1). X-ray fluorescence was collected using a 30-element Ge detector (Canberra). Soller slits with a Z-1 element filter were placed in between the sample chamber and the detector to minimize scattering.

XANES was collected from ± 200 eV relative to the metal edge. The X-ray energy for each metal K_α-edge was internally calibrated to the first inflection point of the Zn foil, 9660.7 eV. EXAFS was collected to 13.5-16 *k* above the edge energy (E_o), depending on the signal:noise at high values of *k*.

The XAS data shown are the average of 7 or 8 scans. Each XANES spectrum used in the average was analyzed for edge energy shifts that might indicate redox chemistry in the beam. None of the samples showed any significant changes. XANES and EXAFS data were analyzed using SixPack.[2] The SixPack fitting software builds on the ifeffit engine[3, 4].

For the EXAFS analysis, each data set was background-corrected and normalized. The data were converted to k-space using the relationship:

$$k = \left[2m_e\left(E - E_o\right)/\hbar^2\right]^{1/2}$$

where m_e is the mass of the electron, $\hbar$ is Plank's constant divided by 2π, and E_o is the threshold energy of the absorption edge. The threshold energy chosen for the Zn was 9670 eV.[1] A Fourier-transform of the data was produced using the data range *k* = 2 - 14 Å^{-1}, where the upper limit was determined by signal:noise. EXAFS was analyzed in r-space over the range r = 1 - 4 Å. Scattering parameters for EXAFS fitting were generated using

FEFF 8.[3] The first coordination sphere was determined by setting the number of scattering atoms in each shell to integer values and systematically varying the combination of N/O- and S-donors.

Multiple-scattering parameters for zinc with histidine imidazole ligands were generated from a previously published crystal structure[5] using the FEFF8 software package[3]. Paths of similar overall lengths were combined to make four imidazole paths[6]. To compare different models of the same data set, ifeffit utilizes three goodness of fit parameters: χ^2, reduced χ^2, and the R-factor. χ^2 is given by equation 1, where N_{idp} is the number of independent data points, N_{ε^2} is the number of uncertainties to minimize, $\mathrm{Re}(f_i)$ is the real part of the EXAFS function, and $\mathrm{Im}(f_i)$ is the imaginary part of the EXAFS fitting function.

$$\chi^2 = \frac{N_{idp}}{N_{\varepsilon^2}} \sum_{i=1}^{N} \left\{ [\mathrm{Re}(f_i)]^2 + [\mathrm{Im}(f_i)]^2 \right\} \qquad (1)$$

Reduced $\chi^2 = \chi^2/(N_{ind} - N_{varys})$, where N_{varys} is the number of refining parameters and represents the degrees of freedom in the fit. Additionally, Ifeffit calculates the R-factor for the fit, which is given by equation 2, and is scaled to the magnitude of the data making it proportional to χ^2.

$$R = \frac{\sum_{i=1}^{N} \left\{ [\mathrm{Re}(f_i)]^2 + [\mathrm{Im}(f_i)]^2 \right\}}{\sum_{i=1}^{N} \left\{ [\mathrm{Re}(\tilde{x}data_i)]^2 + [\mathrm{Im}(\tilde{x}data_i)]^2 \right\}} \qquad (2)$$

In comparing different models, the R-factor and reduced χ^2 parameter were used to determine which model was the best fit for the data. The R-factor will always improve with an increasing number of adjustable parameters, while reduced χ^2 will go through a minimum and then increase, indicating that the model is over-fitting the data7.

Analytical ultracentrifugation. Sedimentation equilibrium data were obtained on a Beckman XL-A Ultracentrifuge using six-cell sample holders (1.2 cm pathlength). Samples of 110 µl *Ec*rmR (2.1 and 5.0 µM in 10 mM HEPES pH 7.5, 150 mM NaCl, 5% glycerol) were centrifuged at 25000 and 35000 rpm (20°C) with a 120 µl buffer blank. Absorbance at 230 0r 276 nm was measured after 10 h and every 2 h thereafter until three consecutive scans overlapped (24 h total) to ensure that equilibrium had been achieved. The partial specific volume for *Ec*FrmR (0.7321 ml g^{-1} at 20°C) was determined from the amino acid content using SEDENTERP[8]. A buffer density of 1.019 g ml^{-1} was also calculated from SEDNTERP. The SEDPHAT software package was used to globally analyze data obtained at multiple rotor speeds and protein concentrations[8].

Supplementary Tables

**Table | S1 Bacterial strains, plasmids and oligonucleotides.**		
	**Relevant characteristics**	**Source**
Strain		
E. coli BL21(DE3)	Controlled expression of T7 RNA polymerase	Laboratory collection
E. coli JRG6703	*E. coli* MG1655 *ΔfrmRAB*	Denby et al. (2015)[9]
E. coli PC677	RZ4500 *ΔfrmRΔlacZ*	This work
E. coli RZ4500	*ΔlacZ*	Choe and Reznikoff (1991)[10]
Plasmid		
pFrmR	pET22b derivative for overproduction of FrmR; Ap^R	This work
pGS2486	pBR322 derivative expressing *frmRAB* from P_{frm}; Ap^R	Denby et al. (2015)[9]
pGS2517	pET22b derivative for overproduction of FrmR(C35A); Ap^R	This work
pGS2518	pET22b derivative for overproduction of FrmR(P2A); Ap^R	This work
pGS2547	pGS2486 derivative expressing *frmR*(P2A); Ap^R	This work
pGS2548	pGS2486 derivative expressing *frmR*(C35A); Ap^R	This work
pJI134	pACYC163 derivative carrying P_{frm}-*frmR-lacZ* fusion; Cm^R	This work
pJI135	pJI134 derivative carrying P_{frm}-*frmR*$_{stop}$-*lacZ* fusion; Cm^R	This work
pJI136	pJI134 derivative carrying P_{frm}-*frmRC35A-lacZ* fusion; Cm^R	This work
pJI156	pJI134 derivative carrying P_{frm}-*frmRH60A-lacZ* fusion; Cm^R	This work
pJI157	pJI134 derivative carrying P_{frm}-*frmRC70A-lacZ* fusion; Cm^R	This work
pJI175	pJI134 derivative carrying P_{frm}-*frmRP2A-lacZ* fusion; Cm^R	This work
pJI177	pJI134 derivative carrying P_{frm}-*frmRA2*-lacZ* fusion; Cm^R	This work
pJI179	pJI134 derivative carrying P_{frm}-*frmRT64A-lacZ* fusion; Cm^R	This work
Oligonucleotide		
JI169	P_{frm}-*frmR-lacZ*; ctatgtcggccgattccttctgccgcccgctatccg	This work
JI170	P_{frm}-*frmR-lacZ*; gtcatagtcgactctcgctcttcctcaatatgg	This work
JI174	pFrmR; ctatgtcatatgcccagtactccggaagagaag	This work
JI175	pFrmR; gtcataccatggctatttaagataggcacgaacc	This work
JI176	*frmR* mutant; gagaagaaaaaggtccttactcgagttcgtcgtattcgggggcagattgatgctct ggaagtgtaggctggagctgcttc	This work
JI177	*frmR* mutant; cagttcaatagtgtcgtcaacggattggctgacttcgcggctgtagcagtcatttcgg tcattccggggatccgtcgacc	This work
JI178	P_{frm}-*frmR*$_{stop}$-*lacZ*; gaaatgcccagtactccgtaagagaagaaaaaggtccttac	This work
JI178r	P_{frm}-*frmR*$_{stop}$-*lacZ*; gtaaggacctttttcttctcttacggagtactgggcatttc	This work
JI179	FrmRC35A; ctggagggtgatgccgaagcccgtgccatactccaacag	This work
JI179r	FrmRC35A; ctgttggagtatggcacgggcttcggcatcaccctccag	This work
JI180	P_{frm} ctatgtgagctctaagccgcggaacaggtgcttac	This work
JI181	P_{frm} gtcataaagcttatctctcgctcttc ctcaatatgg	This work
JI203	FrmRC70A; cgtttgaccgaaatgacgcctacagccgcgaagtcagc	This work
JI203r	FrmRC70A; gctgacttcgcggctgtaggcgtcatttcggtcaaacg	This work
JI310	FrmRA2*; gatgaggtgcgaaatggcgcccagtactccggaag	This work
JI310r	FrmRA2*; cttccggagtactgggcgccatttcgcacctcatc	This work
JI311	FrmRP2A; gatgaggtgcgaaatggccagtactccggaaga	This work
JI311r	FrmRP2A; ctcttccggagtactggccatttcgcacctcatc	This work
C35Af	FrmRC35A; ctgttggagtatggcacgggcttcggcatcaccctccag	This work
C35Ar	FrmRC35A; ctggagggtgatgccgaagcccgtgccatactccaacag	This work
FrmR-BLItz	ggccttccctgccgattag and Biotin-tttcttctcttccggagtactg	This work
FrmR-IVT	gaaattctgattccttctgccgc and cgtttcccggatatggctttcaag	This work

Ndh-IVT	atgcctgatgcgcttcttatca and cgtagtcaacgtaccccc	This work
P2Af	FrmRP2A; cttctcttccggagtactggccatttcgcacctcatcat	This work
P2Ar	FrmRP2A; atgatgaggtgcgaaatggccagtactccggaagagaag	This work
YdhY-BLItz	gttaagggctcagaataatcac and Biotin-ctaatagtggacgatcaaccggg	This work

Table | S2 Selected EXAFS fits for the Zn(II) FrmR complexes.

Sample	*Shell*	*r (Å)*	*σ^2 (x 10^{-3} $Å^{-2}$)*	*ΔE_o (eV)*	*%R*	*Reduced χ^2*
Zn(II)NaCl	2N/O (2Im)	2.08(1)	4(1)	-6(1)	0.92	8.20
	2S	2.307(1)	2.1(3)			
Zn(II)NaBr	2N/O (1Im)	2.00(2)	5(1)	-6(2)	0.47	7.08
	1S	2.27(1)	0(1)			
	1Br	2.44(1)	2.5(5)			

Table | S3 Crystallographic data.

Data collection		
	SeMet SAD FrmR	Formaldehyde-soaked FrmR
Wavelength (Å)	0.9798	0.9763
Resolution range (Å)*	62.58-2.93 (3.01-2.93)	55.25-2.7 (2.83-2.7)
Space group	$P3_112$	$P3_1$
Unit cell (a, b, c, α, β, γ)	144.52,144.52, 56.76<br>90.0, 90.0, 120.0	82.07, 82.07, 55.25,<br>90.0, 90.0, 120.0
Total reflections*	299990 (22859)	60604 (8326)
Unique reflections*	14567 (1083)	11450 (1543)
Multiplicity*	20.6 (21.1)	5.3 (5.4)
Completeness (%)*	99.5 (100.0)	100.0 (100.0)
Mean I/σ (I)*	13.7 (1.7)	12.2 (2.2)
Wilson B factor	62.3	63.5
R_{pim}*	0.048 (0.464)	0.038 (0.499)
Anomalous slope	1.212	-
Anomalous multiplicity*	10.5 (10.7)	-
Anomalous completeness*	99.5 (99.9)	-
Refinement Parameters		*PDB: 5LBM*
R_{factor}		20.9
R_{free}		26.6
Number of non-H atoms		2746
Protein residues		342 (4 chains, A, B, C, D)
RMSD bonds (Å)		0.012
RMSD angles (°)		1.64
Molprobity Score		1.47 (100th percentile N=5412,<br>2.70 Å ± 0.25 Å)
Average B factors ($Å^2$)		
Main chain		76.3
Side chains		84.0
Methylene bridge		90.3

$^aR_{pim} = \Sigma_{hkl} \sqrt{1/n-1}\Sigma_{i=1} | I_i – I_m| / \Sigma_{hkl} \Sigma_i I_i$, where I_i and I_m are the observed intensity and mean intensity of related reflections, respectively. $^bR_{factor}$ = (Σ||Fo|-|Fc||/ Σ |Fo|)*100, where Fo and Fc are observed and calculated structure factor amplitudes. R_{free} is calculated using 5% of reflections omitted from refinement. *Values in parentheses are for highest-resolution shell. Each dataset was collected from a single crystal.

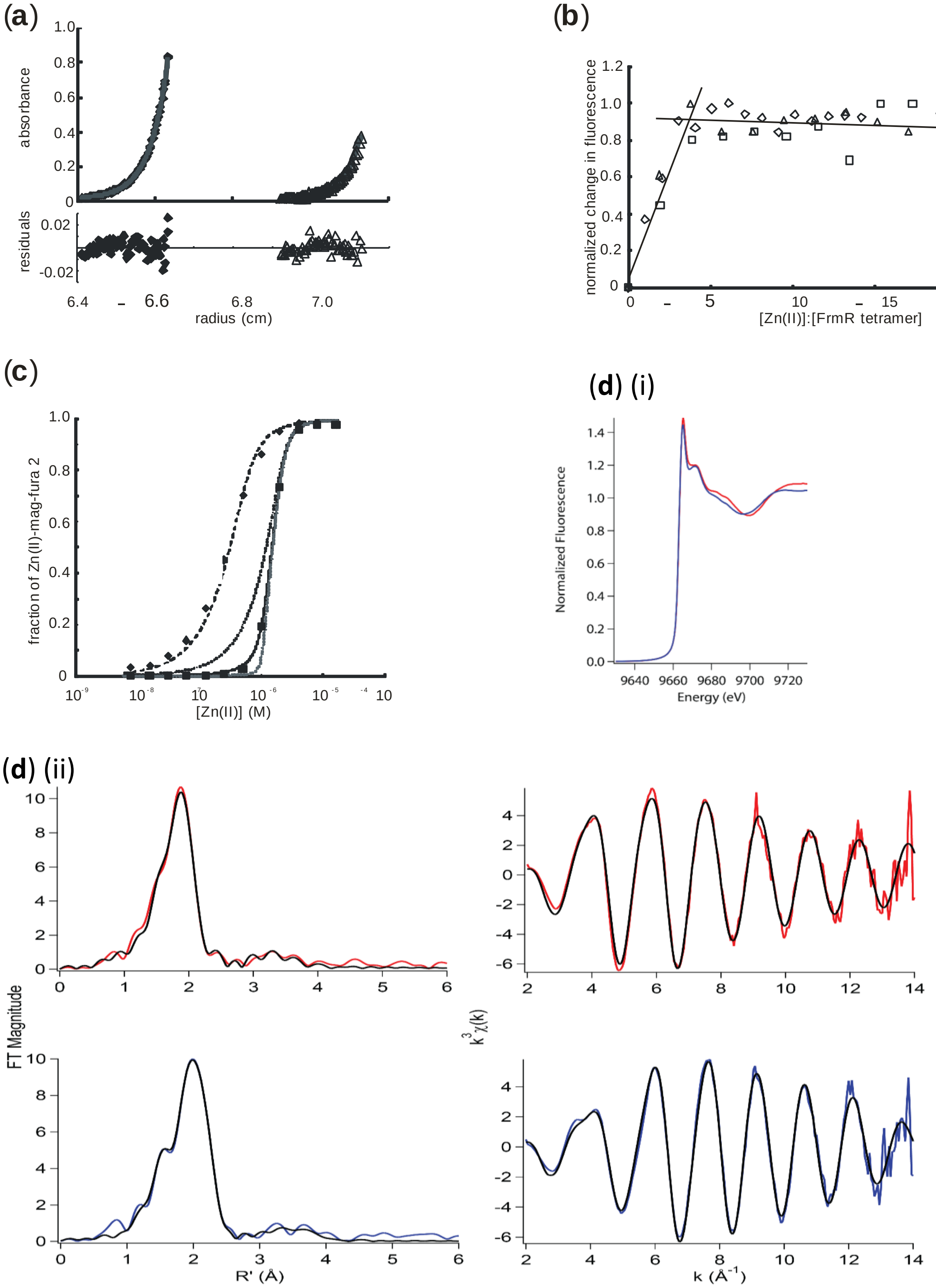

Figure S1 | The *E. coli* FrmR tetramer binds Zn(II). (**a**) Sedimentation equilibrium profiles of FrmR; initial protein concentrations 2.1 μM (open triangles) and 5.0 μM (closed diamonds). The grey lines represent the best fits to the data with residuals shown in the lower panel (see *Methods* for experimental details). (**b**) Changes in intrinsic fluorescence for three titrations (indicated by different symbols) were normalized and plotted against the Zn(II):FrmR tetramer ratio. The solid ines show linear regression fits for the data points during the phase when fluorescence was changing and for the titration end points. (**c**) Zn(II) titrations of the chelator mag-fura 2 (500 nM) (diamonds) and FrmR (0.5 μM tetramer) plus mag-fura 2 (squares). The dashed line is the fit to a single site binding model for mag-fura 2 with a K_d of 61.9 nM. This value was used to obtain the K_{dapp} values for FrmR using a four independent sites binding model (solid line). The dotted lines show the simulated response curves for 10-fold higher and 10-fold lower Zn(II)-binding affinities. Data fits and simulations were done with DYNAFIT[11]. (**d**) X-ray absorption spectroscopy (XAS) of FrmR(Zn(II))$_4$. (i) XANES overlay of *Ec*FrmR(Zn(II)$_4$ in buffer containing either NaCl (red) or NaBr (blue). (ii) Left: Fourier filtered XAS data (colored lines) and best fits (black lines). Right:

Unfiltered k^3-weighted EXAFS spectra and fits.

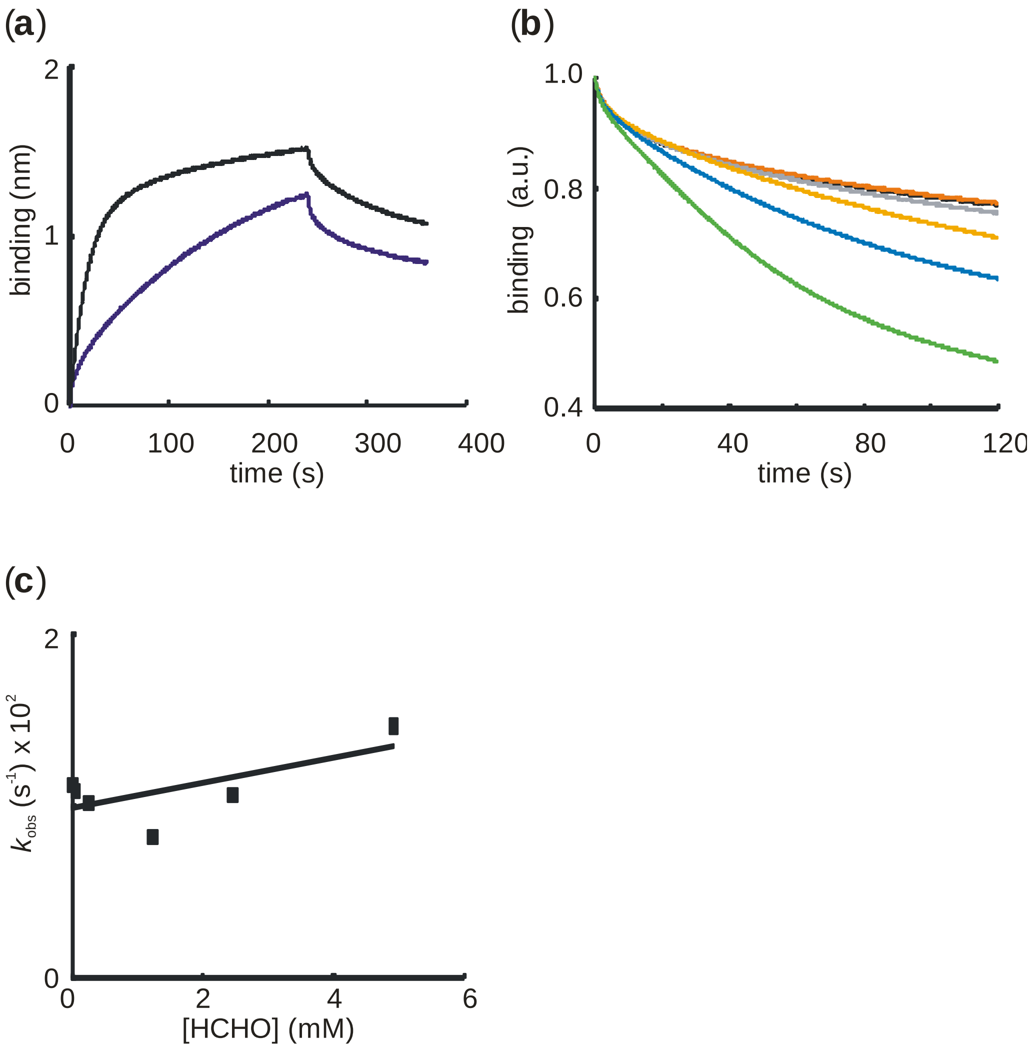

Figure S2 | Zn(II)-loaded FrmR exhibits an impaired response to formaldehyde. (**a**) Bio-Layer Interferometry (BLItz) assays. Traces for interaction of biotin-labeled P_{frm} DNA immobilized on a streptavidin probe with FrmR (2.5 μM tetramer, black line) or FrmR(Zn(II))$_4$ (2.5 μM tetramer; dark blue line) are shown. (**b**) Pre-formed P_{frm}-FrmR(Zn(II))$_4$ complexes were exposed to different concentrations (0, black; 0.05 mM, orange; 0.25 mM, grey; 1.23 mM, yellow; 2.46 mM, blue; 4.92 mM, green) of formaldehyde and disassociation curves were recorded. (**c**) Single exponential fits to the data shown in (**b**) were used to obtain the observed rate constants which were plotted against the concentration of formaldehyde to obtain the apparent second order rate constant.

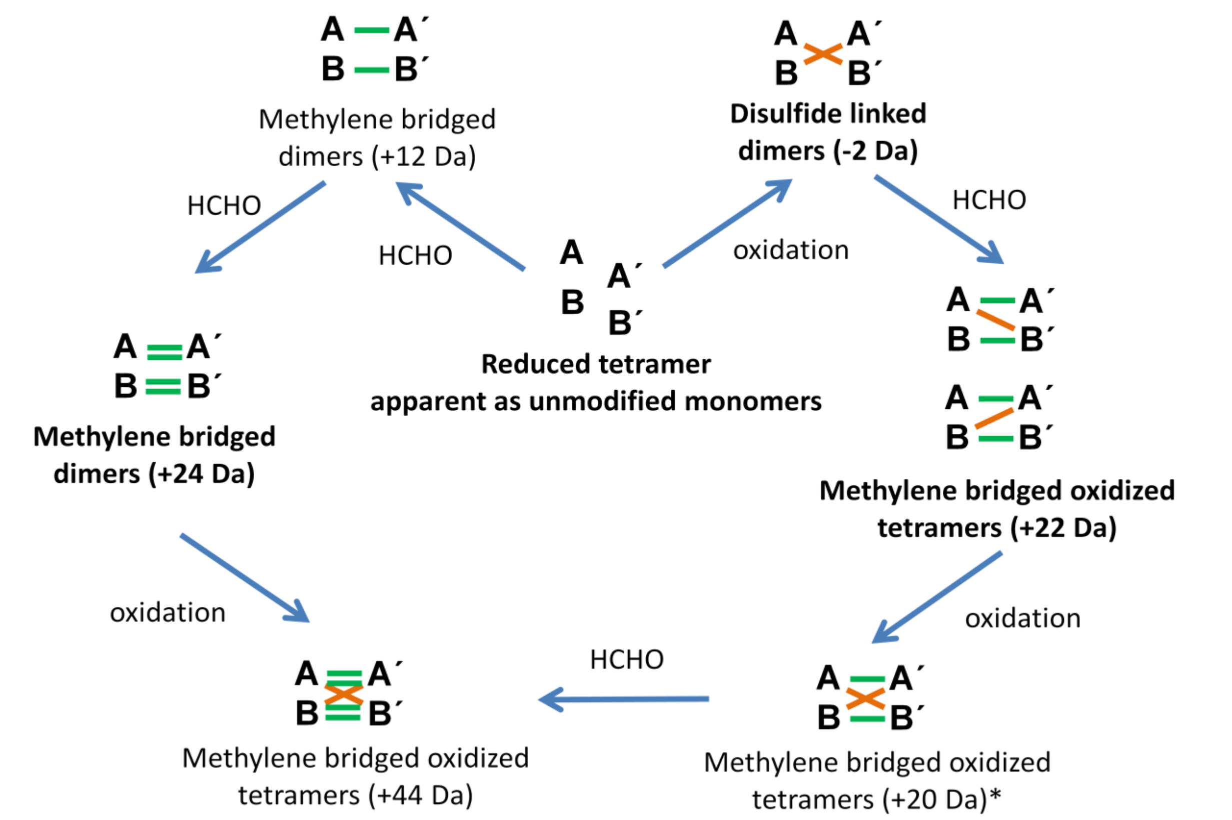

Figure S3 | Schematic of possible species of *Ec*FrmR formed by oxidation and reaction with formaldehyde. The subunits of the *Ec*FrmR tetramer are represented by the upper case bold letters; **A** and **B** on one surface and **A´** and **B´** on the second surface. Disulfide bonds (brown line) and methylene bridges (green lines) are shown linking *Ec*FrmR subunits. Species detected by LC-MS are indicated by bold font along with the approximate predicted mass change compared to the unmodified dimer or tetramer. *Indicates the form of *Ec*FrmR in the crystal structure described here.

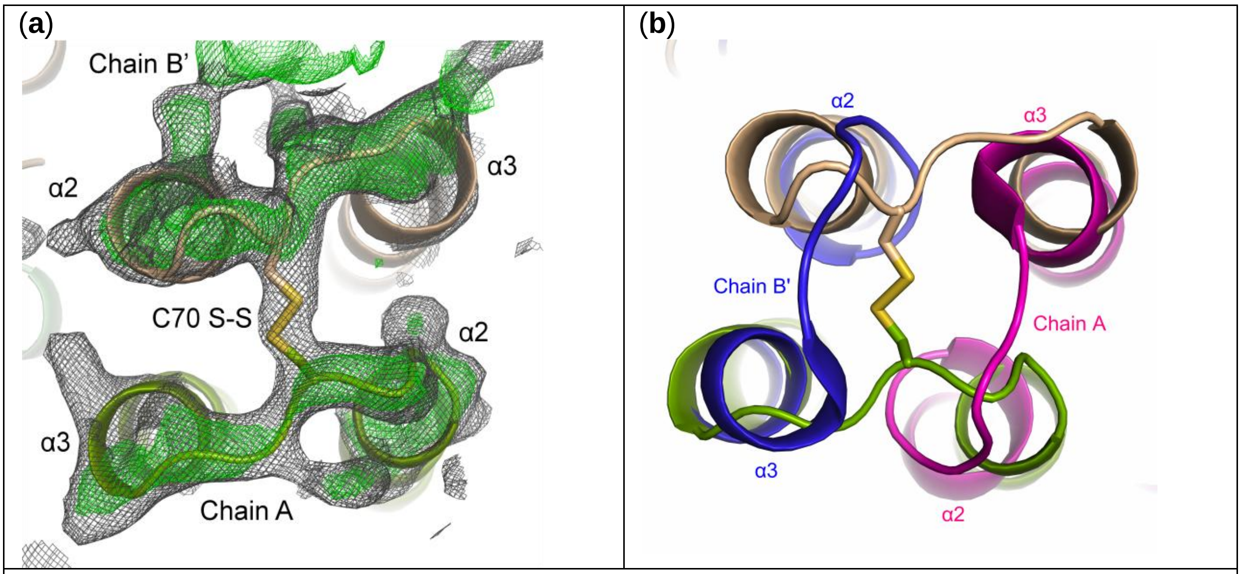

Figure S4 | Section of the *Ec*FrmR structure confirming that the connectivity between α2 and α3 in *Ec*FrmR differs from that in *Sty*FrmR. (**a**) Section of the 2Fo-Fc (black, contoured at 1σ) and 1Fo-Fc (green, contoured at 2.8σ) surrounding the L2 loop region between α2 and α3 of chains B´ (beige) and A (green). The map was generated by extensive refinement of the structure with the coordinates for residues 66-73 of the L2 loop removed from all chains of the model. A cartoon representation of the protein backbone is shown for reference as are the side-chains of Cys70, which form a disulfide bond. (**b**) A superposition between *Ec*FrmR (chain B´, beige and chain A, green) and *Sty*FrmR (chain blue and pink) showing how for *Sty*FrmR the α3 helix is domain swapped onto the opposite face of the tetramer whereas this is not the case for *Ec*FrmR.

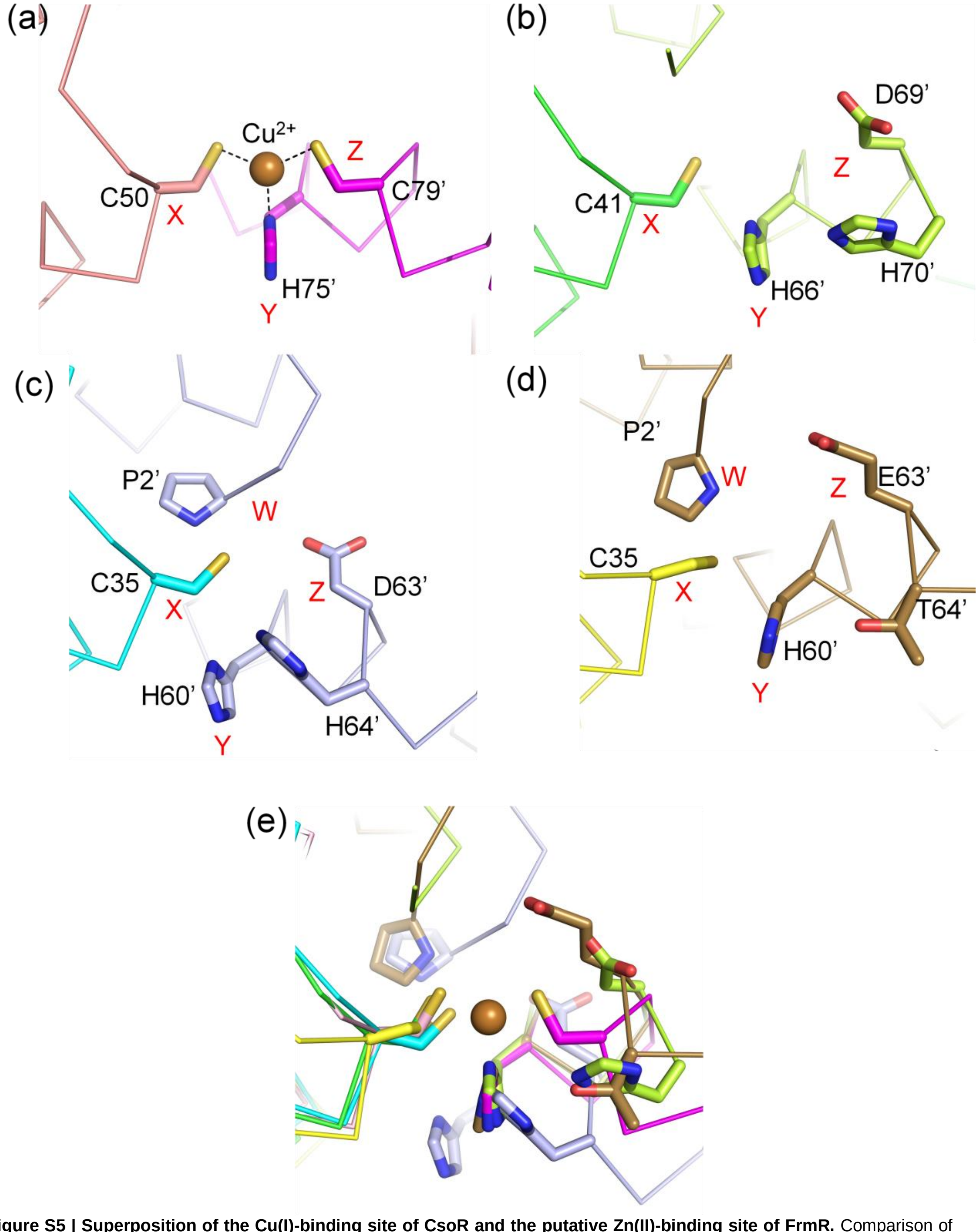

Figure S5 | Superposition of the Cu(I)-binding site of CsoR and the putative Zn(II)-binding site of FrmR. Comparison of the copper-binding sites in (**a**) CsoR from *Geobacillus thermodenitrificans* (PDB: 4M1P[12]; pink) and (**b**) CsoR from *Thermus thermophilus* (PDB: 3AAI[13]; green). The putative Zn(II)-binding sites for (**c**) *Sty*FrmR (PDB: 5LCY[14]; blue) and (**d**) *Ec*FrmR (PDB: 5LBM; yellow); for *Ec*FrmR the subunit with the ordered *N*-terminal region is shown with the cross-link omitted. The four structures are overlaid in (**e**), colored as indicated above, to show the spatial arrangement of the residues around the sites and the relative location of Pro2 (P2´) in FrmR. Amino acid residues are indicated using single letter codes; the W-X-Y-Z fingerprints are indicated by red lettering.

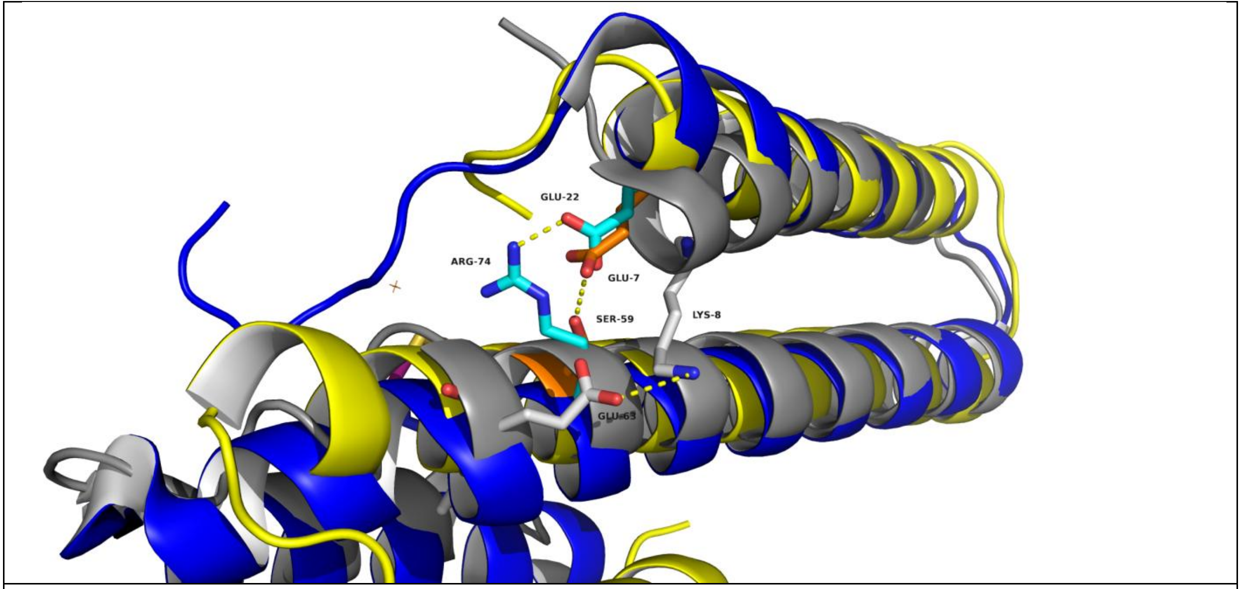

Figure S6 | Signal-dependent interactions between α1 and α2 of three members of the CsoR/RcnR family.
Superposition of the N-terminal region of α1 and the adjacent section of α2 in the signal triggered forms of *Ec*FrmR (yellow helices with orange side chains; PDB: 5LBM), *G. thermodenitrificans* CsoR (blue helices with cyan side chains; PDB: 4M1P) and *M. tuberculosis* CsoR (grey helices and side chains; PDB: 2HH7). Amino acids interacting across the α1-α2 interface are indicated.

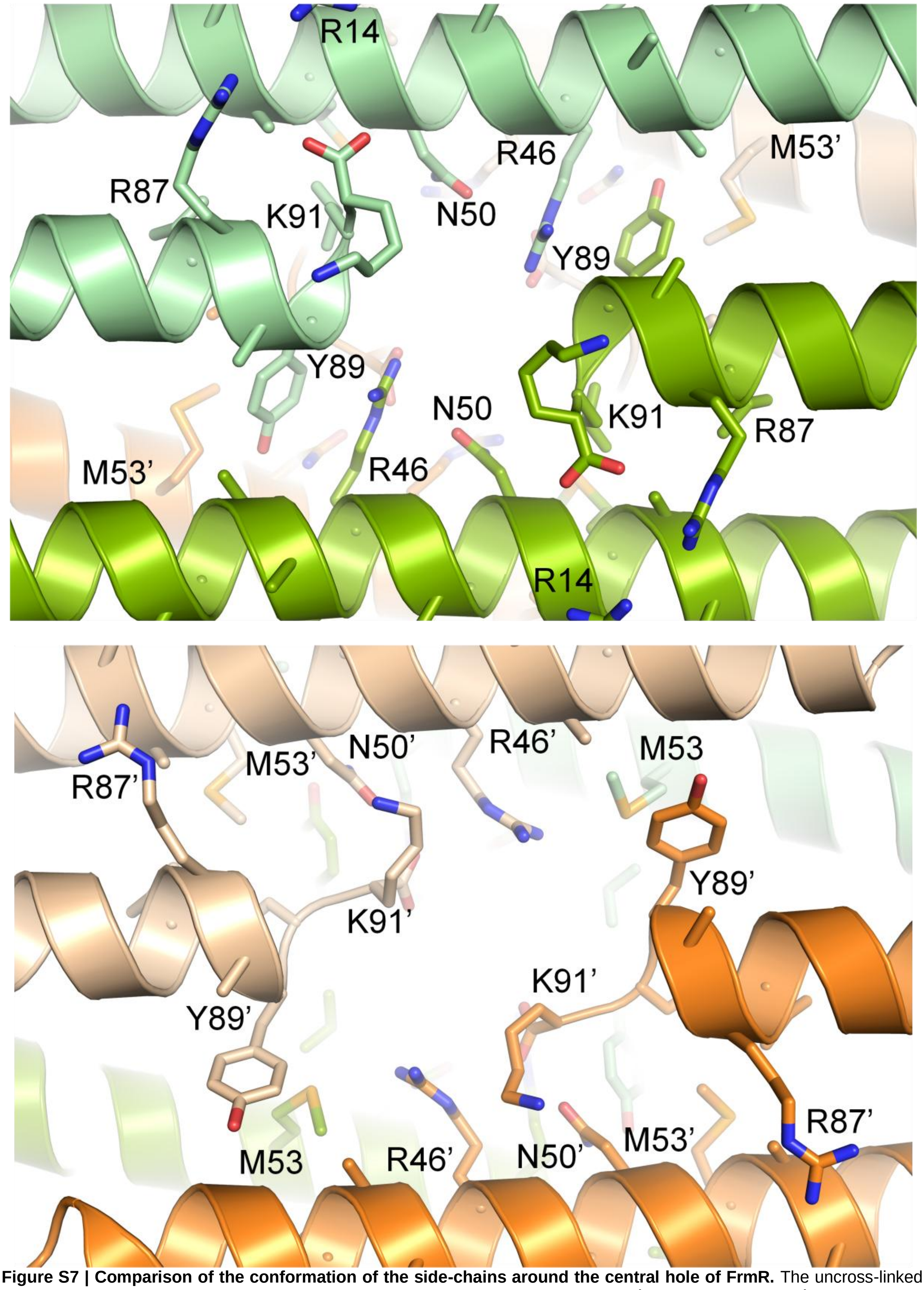

Figure S7 | Comparison of the conformation of the side-chains around the central hole of FrmR. The uncross-linked (green; chain A dark green, chain B light green) and the cross-linked (orange; chain A´ dark orange, chain B´ beige) faces of FrmR are shown. Amino acid residues are indicated using single letter codes.

Supplementary figure legends

Figure S1 | The *E. coli* FrmR tetramer binds Zn(II). (**a**) Sedimentation equilibrium profiles of FrmR; initial protein concentrations 2.1 μM (open triangles) and 5.0 μM (closed diamonds). The grey lines represent the best fits to the data with residuals shown in the lower panel (see *Methods* for experimental details). (**b**) Changes in intrinsic fluorescence for three titrations (indicated by different symbols) were normalized and plotted against the Zn(II):FrmR tetramer ratio. The solid lines show linear regression fits for the data points during the phase when fluorescence was changing and for the titration end points. (**c**) Zn(II) titrations of the chelator mag-fura 2 (500 nM) (diamonds) and FrmR (0.5 μM tetramer) plus mag-fura 2 (squares). The dashed line is the fit to a single site binding model for mag-fura 2 with a K_d of 61.9 nM. This value was used to obtain the K_{dapp} values for FrmR using a four independent sites binding model (solid line). The dotted lines show the simulated response curves for 10-fold higher and 10-fold lower Zn(II)-binding affinities. Data fits and simulations were done with DYNAFIT[15]. (**d**) X-ray absorption spectroscopy (XAS) of $FrmR(Zn(II))_4$. (i) XANES overlay of *Ec*$FrmR(Zn(II)_4$ in buffer containing either NaCl (red) or NaBr (blue). (ii) Left: Fourier filtered XAS data (colored lines) and best fits (black lines). Right: Unfiltered k^3-weighted EXAFS spectra and fits.

Figure S2 | Zn(II)-loaded FrmR exhibits an impaired response to formaldehyde. (**a**) Bio-Layer Interferometry (BLItz) assays. Traces for interaction of biotin-labeled P_{frm} DNA immobilized on a streptavidin probe with FrmR (2.5 μM tetramer, black line) or $FrmR(Zn(II))_4$ (2.5 μM tetramer; dark blue line) are shown. (**b**) Pre-formed P_{frm}-$FrmR(Zn(II))_4$ complexes were exposed to different concentrations (0, black; 0.05 mM, orange; 0.25 mM, grey; 1.23 mM, yellow; 2.46 mM, blue; 4.92 mM, green) of formaldehyde and disassociation curves were recorded. (**c**) Single exponential fits to the data shown in (**b**) were used to obtain the observed rate constants which were plotted against the concentration of formaldehyde to obtain the apparent second order rate constant.

Figure S3 | Schematic of possible species of *Ec*FrmR formed by oxidation and reaction with formaldehyde. The subunits of the *Ec*FrmR tetramer are represented by the upper case bold letters; **A** and **B** on one surface and **A´** and **B´** on the second surface. Disulfide bonds (brown line) and methylene bridges (green lines) are shown linking *Ec*FrmR subunits. Species detected by LC-MS are indicated by bold font along with the approximate predicted mass change compared to the unmodified dimer or tetramer.

Figure S4 | Section of the *Ec*FrmR structure confirming that the connectivity between α2 and α3 in *Ec*FrmR differs from that in *Sty*FrmR. (**a**) Section of the 2Fo-Fc (black, contoured at 1σ) and 1Fo-Fc (green, contoured at 2.8σ) surrounding the L2 loop region between α2 and α3 of chains B´ (beige) and A (green). The map was generated by extensive refinement of the structure with the coordinates for residues 66-73 of the L2 loop removed from all chains of the model. A cartoon representation of the protein backbone is shown for reference as are the side-chains of Cys70, which form a disulfide bond. (**b**) A superposition between *Ec*FrmR (chain B´, beige and chain A, green) and *Sty*FrmR (chain blue and pink) showing how for *Sty*FrmR the α3 helix is domain swapped onto the opposite face of the tetramer whereas this is not the case for *Ec*FrmR.

Figure S5 | Superposition of the Cu(I)-binding site of CsoR and the putative Zn(II)-binding site of FrmR. Comparison of the copper-binding sites in (**a**) CsoR from *Geobacillus thermodenitrificans* (PDB: 4M1P[12]; pink) and (**b**) CsoR from *Thermus thermophilus* (PDB: 3AAI[13]; green). The putative Zn(II)-binding sites in (**c**) StyFrmR (PDB: 5LCY[14]; blue) and (**d**) *Ec*FrmR (PDB: 5LBM; yellow) for *Ec*FrmR the subunit with the ordered *N*-terminal region is shown with the cross-link omitted. The four structures are overlaid in (**e**), colored as indicated above, to show the spatial arrangement of the residues around the sites and the relative location of Pro2 (P2´) in FrmR. Amino acid residues are indicated using single letter codes; the W-X-Y-Z fingerprints are indicated by red lettering.

Figure S6 | Signal-dependent interactions between α1 and α2 of three members of the CsoR/RcnR family. Superposition of the N-terminal region of α1 and the adjacent section of α2 in the signal triggered forms of *Ec*FrmR (yellow helices with orange side chains; PDB: 5LBM), *G. thermodenitrificans* CsoR (blue helices with cyan side chains; PDB: 4M1P) and *M. tuberculosis* CsoR (grey helices and side chains; PDB: 2HH7). Amino acids interacting across the α1-α2 interface are indicated.

Figure S7 | Comparison of the conformation of the side-chains around the central hole of FrmR. The uncross-linked (green; chain A dark green, chain B light green) and the cross-linked (orange; chain A´ dark orange, chain B´ beige) faces of FrmR are shown. Amino acid residues are indicated using single letter codes.

Supplementary animation

An animation showing the conformational changes that occur when formaldehyde-induced methylene bridges are formed on one face (orange and beige subunits) of the

***E. coli* FrmR tetramer.** The subunits forming the uncross-linked surface are shown in light and dark green. Pro2 and Cys35 are shown as space-fill representations in atom colors (blue, N; yellow, S). Transitions between the uncross-linked and cross-linked states were generated by morphing between the known conformations of the uncross-linked and cross-linked coordinates in Lsqman[15], before producing the animation in PyMOL[16].

References for supplementary data

1. Leitch, S., Bradley, M. J., Rowe, J. L., Chivers, P. T.& Maroney, M. J. Nickel-specific response in the transcriptional regulator, *Escherichia coli* NikR. *J. Am. Chem. Soc.* 129, 5085-5095 (2007).

2. Webb, S. M. SIXpack: a graphical user interface for XAS analysis using IFEFFIT. *Physica Scripta* T115, 1011-1014 (2005).

3. Ankudinov, A. L., Ravel, B., Rehr, J. J. & Conradson, S. D. Real-space multiple-scattering calculation and interpretation of x-ray-absorption near-edge structure. *Physic. Rev. B* 58, 7565-7576 (1998).

4. Zabinsky, S. I., Rehr, J. J., Ankudinov, A., Albers, R. C. & Eller, M. J. Multiple-scattering calculations of x-ray-absorption spectra. *Physic. Rev. B Condensed Matter* 52, 2995-3009 (1995).

5. Gasper, R., Scrima, A.& Wittinghofer, A. Structural insights into HypB, a GTP-binding protein that regulates metal binding. *J. Biol. Chem.* 281, 27492-27502 (2006).

6. Costello, A., Periyannan, G., Yang, K. W., Crowder, M. W. & Tierney, D. L. Site-selective binding of Zn(II) to metallo-beta-lactamase L1 from *Stenotrophomonas maltophilia*, *J.Biol. Inorg. Chem.* 11, 351-358 (2006).

7. Herbst, R. W., Guce, A., Bryngelson, P. A., Higgins, K. A., Ryan, K. C., Cabelli, D. E., Garman, S. C. & Maroney, M. J. Role of conserved tyrosine residues in NiSOD catalysis: a case of convergent evolution, *Biochem.* 48, 3354-3369 (2009).

8. Lebowitz, J., Lewis, M. S. & Schuck, P. Modern analytical ultracentrifugation in protein science: a tutorial review. *Protein Sci.* **11**, 2067-2079 (2002).

9. Denby, K. J., Rolfe, M. D., Crick, E., Sanguinetti, G., Poole, R. K. & Green, J. Adaptation of anaerobic cultures of *Escherichia coli* K-12 in response to environmental trimethylamine-N-oxide. *Environ. Microbiol.* **17**, 2477-2491 (2015).

10. Choe, M. & Reznikoff, W. S. Anaerobically expressed *Escherichia coli* genes identified by operon fusion techniques. *J. Bacteriol.* **173,** 6139-6146 (1991).

11. Kuzmic, P. DynaFit – A software package for enzymology. *Meth. Enzymol.* **467**, 247–280 (2009).

12. Chang, F. M., Coyne, H. J., Cubillas, C., Vinuesa, P., Fang, X., Ma, Z., Ma, D., Helmann, J. D., García-de los Santos, A., Wang, Y. X. et. al. Cu(I)-mediated allosteric switching in a copper-sensing operon repressor (CsoR). *J. Biol. Chem.* **289**, 19204-19217 (2014).

13. Sakamoto, K., Agari, Y., Agari, K., Kuramitsu, S. & Shinkai, A. Structural and functional characterization of the transcriptional repressor CsoR from *Thermus thermophilus* HB8. *Microbiol.* **156**, 1993-2005 (2010).

14. Osman, D., Piergentili, C., Chen, J., Sayer, L. N., Uson, I., Huggins, T. G., Robinson, N. J. & Pohl, E. The effectors and sensory sites of formaldehyde-responsive regulator FrmR and metal-sensing variant. *J. Biol. Chem.* doi:10.1074/jbc.M116.745174 (2016).

15. Kleywegt, G. J. Use of non-crystallographic symmetry in protein structure refinement. *Acta Crystallogr. Sect. D* **52**, 842-857 (1996).

16. DeLano, W. L. The PyMOL Molecular Graphics System, Version 1.8 Schrödinger, LLC *DeLano Scientific, San Carlos, CA, USA* (2002).
